# A systematic review of existing ageism scales

**DOI:** 10.1016/j.arr.2019.100919

**Published:** 2019-09

**Authors:** Liat Ayalon, Pnina Dolberg, Sarmitė Mikulionienė, Jolanta Perek-Białas, Gražina Rapolienė, Justyna Stypinska, Monika Willińska, Vânia de la Fuente-Núñez

**Affiliations:** a Louis and Gabi Weisfeld School of Social Work, Bar Ilan University, Ramat Gan, 52900, Israel; b Ruppin Academic Center, Emek Hefer, 4025000, Israel; c Institute of Sociology, Lithuanian Social Research Centre, Vilnius, LT-01108, Lithuania; d Institute of Sociology and Center of Evaluation and Public Policy Analysis, Jagiellonian University in Cracow, Cracow, 31-004, Poland; e Institute of Sociology, Lithuanian Social Research Centre, Vilnius, LT-01108, Lithuania; f Free University Berlin, Institute for East European Studies, Department of Sociology, Berlin, 14195, Germany; g School of Health and Welfare, Jönköping University, 551 11, Jönköping, Sweden; h Department on Ageing and Life Course, World Health Organization, 20 Avenue Appia, Geneva, 1221, Switzerland

**Keywords:** Ageism, Scale, Discrimination, Stereotype, Prejudice, Systematic review

## Abstract

Ageism has been shown to have a negative impact on older people’s health and wellbeing. Though multiple scales are currently being used to measure this increasingly important issue, syntheses of the psychometric properties of these scales are unavailable. This means that existing estimates of ageism prevalence may not be accurate. We conducted a systematic review aimed at identifying available ageism scales and evaluating their scope and psychometric properties. A comprehensive search strategy was used across fourteen different databases, including PubMed and CINAHL. Independent reviewers extracted data and appraised risk of bias following the COnsensus-based Standards for the selection of health Measurement INstruments (COSMIN) guidelines. Of the 29,664 records identified, 106 studies, assessing 11 explicit scales of ageism, were eligible for inclusion. Only one scale, the ‘Expectations Regarding Aging’ met minimum requirements for psychometric validation (i.e., adequate content validity, structural validity and internal consistency). Still, this scale only assesses the ‘stereotype’ dimension of ageism, thus failing to evaluate the other two ageism dimensions (prejudice and discrimination). This paper highlights the need to develop and validate a scale that accounts for the multidimensional nature of ageism. Having a scale that can accurately measure ageism prevalence is key in a time of increasing and rapid population ageing, where the magnitude of this phenomenon may be increasing.

## Highlights

• There is a general lack of psychometric assessments on existing ageism scales.
• None of the available ageism scales have both adequate scope and psychometric validity.
• Current estimates of ageism incidence and prevalence may not be accurate.

## 1 Introduction

Ageism is increasingly recognized as a public health issue and as one of the most prevalent forms of stereotyping, prejudice and discrimination (World Health Organization, 2015). Although ageism can affect any age group, existing evidence suggests that older people are at higher risk of suffering from its negative consequences. Indeed, ageist beliefs and attitudes have been shown to impair older people’s cognitive and functional performance (Lamont et al., 2015), result in poorer mental health (Wurm and Benyamini, 2014), increased morbidity (Allen, 2015) and poorer recovery from disability (Levy et al., 2012). Ageism is also associated with a shorter lifespan (Levy et al., 2002) and feelings of distress and loneliness (McHugh, 2003), and can result in the marginalization of older adults (Vitman et al., 2013) as well as their exclusion from meaningful roles in society (Wethington et al., 2016). In a time of increasing and rapid population ageing, it is possible that the prevalence of ageism against older adults is increasing. However, existing knowledge about the measures used to estimate its magnitude and prevalence is rather limited (Officer and de la Fuente-Núñez, 2018; Wilson et al., 2019). Hence, existing estimates of ageism prevalence may not be accurate.

Ageism is considered to include three dimensions: stereotypes (cognitive component - e.g., I *think* older adults are a burden to society); prejudice (emotional component - e.g., I do not *enjoy* conversations with older adults); and discrimination (behavioral component - e.g., I try not to *interact* with older adults) (Iversen et al., 2009). It can be directed towards others (e.g., I enjoy telling jokes *about older adults*) or towards oneself (e.g., I am concerned about *my own aging*) (Ayalon and Tesch-Römer, 2017), it can be positive (e.g. the stereotype that older adults are wise) or negative (e.g. the stereotype that older adults are slow), and it can be explicit (conscious) or implicit (unconscious) (Levy and Banaji, 2002). The extent to which these various dimensions and facets of ageism are reflected in existing ageism scales is not known. Our understanding of what existing ageism scales measure is further compounded by the lack of standard and operational definitions in the field, especially around the concept of “older person”. For example, ageism scales often include terms like “old people” (e.g. “I enjoy being around old people”) without providing clear indications as to what this term refers to. The use of age cut-offs to define different age groups could be an option to resolve this challenge though it comes with its own issues. Age categories or groups are socially defined, so selecting one age cut-off over another is inevitably arbitrary and may not be equally relevant across different contexts. We also lack systematic knowledge about the psychometric properties of available scales. Thus, it is currently unclear what existing ageism scales measure and what psychometric quality they have.

To address these gaps, we conducted the first ever systematic review aimed at identifying available scales of ageism against older adults and evaluating their scope and psychometric properties. This knowledge is essential for the identification of comprehensive and psychometrically valid scales that can be efficiently used to map out different aspects of ageism and its prevalence. It can also serve as an important reference point when assessing if and how available strategies developed to reduce or prevent ageism work.

## 2 Methods

### 2.1 Search strategy and selection criteria

We conducted a systematic review in accordance with the Preferred Reporting Items for Systematic Reviews and Meta-Analyses (PRISMA) guidelines and following a protocol that was registered in PROSPERO (ID: CRD42018087371). The results of all searches were entered into the Covidence software programme for systematic reviews (Covidence, 2017). A comprehensive search string on ‘ageism’ was developed for PubMed and subsequently ‘translated’ for searches in 13 additional electronic databases up until December 2017 (see Appendix A). Following an initial phase of removing duplicates and completely irrelevant records, titles/abstracts were screened to determine inclusion by at least two independent raters among the authors. Records were divided randomly across reviewers, and disagreements were resolved through consensus with a third reviewer.

A snowball search was conducted to identify additional records for full-text review by using Google Scholar’s “related to” and “cited by” functions for each of the articles included in the original search (Atkinson and Cipriani, 2018). To ensure comprehensiveness, a *specific search* of articles mentioning the scales identified in the initial round was also conducted in selected databases (EMBASE, Web of Science, EBSCO). The bibliographies of the final set of records were also reviewed for the identification of additional articles. Full-text review was performed independently by at least two raters, who resolved disagreements through consensus with a third reviewer (among LA, VFN, MW, JPB).

Eligible studies met the following inclusion criteria: a) available in English, Spanish or French; b) published between 1970 (as the term ‘ageism’ was coined in 1969) and 2017; c) aimed to develop/evaluate measurement properties of a quantitative scale of ageism against older adults; d) presented original research; e) assessed an ageism scale that was evaluated by at least two additional independent research groups. The rationale for this last criterion is that a minimum number of independent studies are needed for the psychometric validation of a scale. This criterion was also applied to cases where significantly different scales used the same reference name (e.g. the implicit association test, which covers a wide range of methodologies and content). In addition, studies that assessed a subscale of ageism, rather than a whole scale, were excluded.

### 2.2 Data extraction and quality assessment

The data extraction and risk of bias tool was adapted from the COnsensus-based Standards for the selection of health Measurement INstruments (COSMIN) guidelines (Mokkink et al., 2018; Terwee et al., 2018), piloted and refined before extraction. Four main categories of data were extracted: study characteristics, scale characteristics, quality of measurement properties and methodological quality of measurement properties per study. Nine psychometric properties were assessed for each scale: content validity, structural validity, internal consistency, cross-cultural validity, reliability, measurement error, criterion validity, construct validity, responsiveness (details provided in Supplementary Appendix A). The COSMIN guidelines were used to evaluate both the measurement properties of each scale (adequate (+), inadequate (-) or indeterminate (?)), and the methodological quality of each measurement property per study (Very good, Adequate, Doubtful, Inadequate) (COSMIN criteria provided in Supplementary Appendix B).

Two independent, randomly assigned raters among the authors extracted data from each included record and appraised risk of bias for each psychometric property per study. Disagreements were resolved through consensus with a third reviewer (LA or VFN).

### 2.3 Data analysis

Two independent raters (LA, VFN) appraised the overall rating for each psychometric property and the overall quality of the body of evidence for each measurement property of a given scale, following the COSMIN guidelines (Mokkink et al., 2018; Terwee et al., 2018). The overall rating of each psychometric property per scale could be either sufficient (+), insufficient (-), indeterminate (?) or inconsistent (±) and would depend on the scores obtained across individual studies. For example, if a given measurement property was graded as insufficient in most of the individual studies, then the overall rating would be insufficient. Indeterminate (?) ratings given for individual studies were disregarded when determining the overall quality of a measurement property of a scale unless the measurement property had no ‘sufficient’ or ‘insufficient’ ratings across individual studies. As COSMIN guidelines require reviewers to conduct their own assessment of the content validity of each scale, indeterminate (?) overall ratings for content validity are never possible. To evaluate the overall rating for content validity, we independently assessed the face validity of each scale (LA, VFN). This evaluation was conducted based on the first published version and the first proposed division into factors or subscales. To form our judgement, we examined whether the items included in the scale were relevant to the concept of ageism, whether the items included under each subscale fell into a cohesive domain, and whether the phrasing of the items and the instructions were easy to understand (Terwee et al., 2018).

The overall quality of the body of evidence per psychometric property was downgraded on three accounts: risk of bias, inconsistency of findings across individual studies and imprecision (see Supplementary Appendix C). A fourth factor, indirectness, was not considered relevant in this review because the ageism scales included in the study did not have a clearly defined target population. For structural validity and internal consistency, we also downgraded the overall quality of the evidence if substantial variations in the number of items and factors used for the same scale were evident across studies, as we considered this part of inconsistency.

In interpreting the findings, the presence of adequate content validity, structural validity and internal validity were considered as minimal criteria to support the psychometric validation of a scale (Mokkink et al., 2018).

## 3 Results

**Fig. 1:**
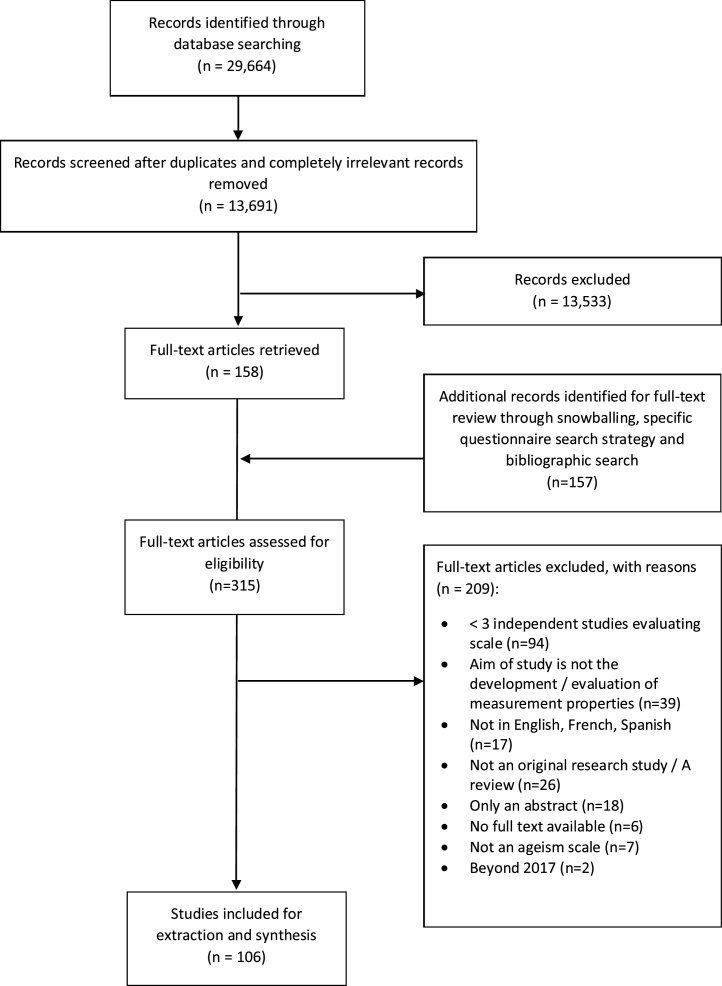
Study selection.

A total of 29,664 records were retrieved based on the original search. Of these, 158 were identified for full-text review following removal of duplicates and irrelevant records. An additional 157 records were identified for full-text review via snowballing, the specific search strategy and bibliographic searches. Of these 315 records, 209 were excluded (See Fig. 1). This resulted in 106 records included in this systematic review, which assessed 11 scales aimed at measuring explicit ageism. Table 1 reports the number of studies looking into each individual scale. Details on the characteristics of the different studies included in the review, and the characteristics of the 11 scales are provided in Supplementary Table 1 and Supplementary Table 2, respectively. It is worth highlighting that most studies were conducted in English speaking, high-income countries, including Australia, Canada and the United States of America, and that no studies were conducted in low and lower-middle income countries. It is also worth noting that participants in these studies were mainly older adults or university students. Information on the psychometric properties of each scale per individual study and on the quality of the evidence for each psychometric property per individual study is available upon request.

**Table 1:** Number of studies per scale.

Scale name	Dimension(s) of ageism assessed	No. of articles	References
Aging perceptions questionnaire	Explicit: Stereotypes, prejudice, discrimination	7	(Barker et al., 2007; Ingrand et al., 2012; Sexton et al., 2014; Slotman et al., 2015; Chen et al., 2016; Moghadam et al., 2016; Slotman et al., 2017)
Aging semantic differential	Explicit: Stereotypes	15	(Rosencranz and McNevin, 1969; Underwood et al., 1985; Gekoski et al., 1991; O’Hanlon et al., 1993; Intrieri et al., 1995; Villar Posada, 1997; Polizzi and Millikin, 2002; Polizzi, 2003; Stewart et al., 2007; Iwasaki and Jones, 2008; Boudjemad and Gana, 2009; Gluth et al., 2010; Gonzales et al., 2010; Carlson, 2015; Gonzales et al., 2017)
Anxiety about ageing questionnaire	Explicit: Stereotypes, prejudice, discrimination	6	(Lasher, 1987; Watkins et al., 1998; Rivera-Ledesma et al., 2007; Gao, 2012; Koukouli et al., 2013; Sargent-Cox et al., 2014)
Attitudes to aging questionnaire	Explicit: Stereotypes, prejudice, discrimination	9	(Laidlaw et al., 2007; Chachamovich et al., 2008; Kalfoss et al., 2010; Lucas-Carrasco et al., 2013; Shenkin et al., 2014; Brown et al., 2015; Helmes and Pachana, 2016; Marquet et al., 2016; Rejeh et al., 2017)
Expectations Regarding Aging	Explicit: Stereotypes	6	(Sarkisian et al., 2001, 2002; Sarkisian et al., 2005; Joshi et al., 2010; Beser et al., 2012; Sparks et al., 2013)
Facts on aging quiz	Explicit: Stereotypes	35	(Palmore, 1977; Klemmack, 1978; Holtzman and Beck, 1979; Miller and Dodder, 1980; Laner, 1981; Luszcz, 1982; Romeis and Sussman, 1982; Matthews et al., 1984; Miller and Dodder, 1984; Courtenay and Weidemann, 1985; Dail and Johnson, 1983, 1986; McCutcheon, 1986; Donnelly et al., 1987; Norris et al., 1987; Kline et al., 1990; Kline and Kline, 1991a, b; O’Hanlon et al., 1993; Lusk et al., 1995; Harris and Changas, 1994; Harris et al., 1996; Kramer et al., 2001; Pennington et al., 2001; Obiekwe, 2001; Seufert and Carrozza, 2002; Cowan et al., 2004; Runkawatt, 2007; Unwin et al., 2008; Wang et al., 2010; Nakao et al., 2013; Pachana et al., 2013; Van der Elst et al., 2014; Helmes, 2016; Helmes and Pachana, 2016; Shiovitz-Ezra et al., 2016)
Fraboni scale of ageism	Explicit: Stereotypes, prejudice, discrimination	8	(Fraboni et al., 1990; Rupp et al., 2005; Bodner and Lazar, 2008; Boudjemad and Gana, 2009; Lin et al., 2010; Kutlu et al., 2012; Helmes and Pachana, 2016; Shiovitz-Ezra et al., 2016)
Image of aging scale	Explicit: Stereotypes	3	(Levy et al., 2004; Bai et al., 2012; Fernández-Ballesteros et al., 2017)
Kogan’s attitudes towards older people scale	Explicit: Stereotypes, prejudice	17	(Kogan, 1961; Hicks et al., 1976; Wingard, 1980; Hilt, 1997; Söderhamn et al., 2000; Lambrinou et al., 2005; Ogiwara et al., 2007; Runkawatt, 2007; Iwasaki and Jones, 2008; Helmes and Campbell, 2009; Yen et al., 2009; Erdemir et al., 2011; Küçükgüçlü et al., 2011; Kiliç and Adibelli, 2011; Rejeh et al., 2012; Matarese et al., 2013; Vitman-Schorr et al., 2014)
Reactions to aging questionnaire	Explicit: Stereotypes, prejudice	5	(Gething, 1994; Netz et al., 2001; Getting et al., 2002; Gething et al., 2004; Helmes and Pachana, 2016)
Tuckman and Lorge questionnaire	Explicit: Stereotypes	6	(Tuckman and Lorge, 1954; Axelrod and Eisdorfer, 1961; Eisdorfer, 1966; Hicks et al., 1976; Wingard, 1980; Helmes and Campbell, 2009)

Note: Some articles evaluated more than one scale.

Table 2 reports the aggregated rating of the psychometric properties of each scale, as well as the overall quality of the evidence for each measurement property across studies. Of the included scales, only one, the Expectations Regarding Aging Questionnaire, had adequate ratings on the three psychometric properties that are considered indispensable for psychometric validation (content validity, structural validity, internal consistency). This scale was assessed in six studies. Its longer version consists of 38 items and two subscales (Sarkisian et al., 2002) whereas the shorter version consists of 12 items along three subscales (Sarkisian et al., 2005). The scale examines stereotypes towards old age in general (“When people get older, they need to lower their expectations of how healthy they can be”) and towards oneself (“I expect that as I get older, I will get tired more quickly”). The response scale offers four options (1-definitely true, 4-definitely false).

**Table 2:** Overall rating of each measurement property per scale and grading of the quality of evidence per measurement property per scale across all studies.

		Aging perceptions questionnaire	Aging semantic differential	Anxiety about ageing questionnaire	Attitudes to aging questionnaire	Expectations Regarding Aging	Facts on aging quiz	Fraboni scale of ageism	Image of aging scale	Kogan’s attitudes towards older people scale	Reactions to aging questionnaire	Tuckman & Lorge Questionnaire
**Content validity**	Overall rating	+/-	+	+/-	+/-	+	+/-	+/-	+	+/-	+/-	+/-
	Quality of evidence	Moderate	Moderate	Low	Moderate	Moderate	Moderate	Moderate	Moderate	Moderate	Moderate	Very low
**Structural validity**	Overall rating	+	–	+	+	+	+	+	+/-	+	?	?
	Quality of evidence	Low	Low	Moderate	Low	Moderate	Moderate	Moderate	Low	Low	Moderate	Very low
**Internal consistency**	Overall rating	–	+	–	+	+	?	–	+/-	+	–	?
	Quality of evidence	Low	Low	Low	Low	Low	Low	Low	Low	Low	Moderate	Low
**Cross cultural validity/measurement invariance**	Overall rating	–	+	–	–	?	?	+	–		–	
	Quality of evidence	Low	Moderate	Low	Moderate	Very low	Moderate	Low	Moderate		Very low	
**Reliability**	Overall rating	+	+		+/-	+	–	+	+	+		–
	Quality of evidence	Moderate	Moderate		Moderate	Moderate	Moderate	Very low	Moderate	Moderate		Moderate
**Measurement error**	Overall rating						?					
	Quality of evidence						Very low					
**Criterion validity**	Overall rating	+				?				?		
	Quality of evidence	High				Moderate				Very low		
**Construct validity**	Overall rating	+ (convergent & known groups)	+(convergent) +/- (known groups)	+(convergent) - (known groups)	+ (convergent & known groups)	+ (convergent & known groups)	-(convergent), + (known groups)	+ (convergent & known groups)	+ (convergent & known groups)	+/-(convergent), + (known groups)	+/-(convergent),? (known groups)	?(convergent)+ (known groups)
	Quality of evidence	High (convergent), Moderate (known groups)	Low (convergent & known groups)	Moderate (convergent & known groups)	Low (convergent), Moderate (known groups)	Moderate (convergent), Low (known groups)	Low (convergent & known groups)	Moderate (convergent & known groups)	High (convergent), Low (known groups)	Low (convergent & known groups)	Low (convergent), Moderate (known groups)	Very low (convergent), Moderate (known groups)
**Responsiveness**	Overall rating		?				+					?
	Quality of evidence		Very low				Very low					Very low

*Overall rating of psychometric property*: + (sufficient), - (insufficient), +/- (inconsistent),? (indeterminate). *Overall quality of the evidence*: High (very confident that the true measurement lies close to the estimate), Moderate (moderately confident in the measurement property estimate), Low (limited confidence in the measurement property), Very low (very little confidence in the measurement property estimate).

Content validity was judged as adequate as included items were easy to understand and were adequately distributed into two subscales, one measuring self-expectations and the other measuring general expectations regarding ageing. Structural validity, internal consistency, reliability, and construct validity (known groups, convergent) were also judged as adequate. The quality of the evidence ranged between moderate (content validity, structural validity, reliability, convergent validity), low (internal consistency, known groups), and very low (cross cultural validity).

Two additional scales met two of the three minimum criteria for psychometric validation (structural validity and internal consistency) and may benefit from further modifications to resolve the current inconsistent rating (±) for content validity. The first is the Attitudes to Aging Questionnaire, which was assessed by nine different studies (with some studies conducted by the same group and possibly using overlapping data (Laidlaw et al., 2018, 2007; Lucas-Carrasco et al., 2013; Shenkin et al., 2014)). It consists of 24 statements about old age divided into three subscales, and ranked on a 5-point Likert scale (Laidlaw et al., 2007). The scale examines stereotypes, prejudice and discrimination towards others and towards oneself. Content validity was judged as inconsistent (±) because the proposed factor structure included, under the same factor, items that assess both stereotypes towards others (“old age is a time of loneliness”) and towards oneself (“I am losing my physical independence as I get older”). Some items seemed ambiguous with regards to the age group concerned (e.g. “I feel excluded from things because of my age”), and others seemed to be only appropriate for people over a certain age (e.g. “I don’t feel involved in society now that I’m older”). The quality of the evidence for this property was moderate. Structural validity, internal consistency, and construct validity (convergent and known-groups validity) were judged as adequate, with the quality of the evidence being rated as low across all measurement properties, except for known-groups validity, which was rated as moderate. Reliability was indeterminate and cross-cultural validity was inadequate with the quality of the evidence being rated as moderate for both properties.

The second scale that met two of the three minimum criteria for psychometric validation was the Kogan’s Attitudes towards Old People Scale, which was evaluated by 17 different studies (with two of the studies being based on the same data (Hicks et al., 1976; Wingard, 1980)). This is a 34-item scale, composed of a positive subscale including 17 items (e.g., “it is evident that most old people are very different from one another”) and a negative subscale including 17 items (e.g., “old people have too much power in business and politics”). The scale assesses explicit prejudice and stereotypes towards older people using a 7-point Likert response scale. The content validity of this scale was rated as inconsistent (±) because the proposed factor structure fails to consider the two dimensions of ageism that are being assessed - prejudice and stereotypes. For example, an item assessing prejudice (“If old people expect to be liked, their first step is to try to get rid of their irritating faults”) is included under the same factor as an item assessing stereotypes (“Most old people are constantly complaining about the behavior of the younger generation”). Also, though the two proposed factors are supposed to include identical items phrased either positively or negatively, this is not always the case. For example, the negative factor includes the item “If old people expect to be liked, their first step is to try to get rid of their irritating faults” in opposition to the item “When you think about it, old people have the same faults as anybody else.” Some items are also difficult to understand (e.g. “…it’s hard to figure out what makes them tick”). The overall quality of the evidence for this property was moderate. Structural validity, internal consistency, reliability and known groups validity were rated as adequate and their evidence base was rated as low, except for reliability which was rated as moderate. Convergent validity was rated as inconsistent and criterion validity as indeterminate (?) with the quality of evidence being low and very low, respectively.

## 4 Discussion

Identifying a comprehensive scale with adequate psychometric properties is a necessary step in tackling ageism. As past research in the field of ageism has largely relied on scales that have not been comprehensively evaluated for psychometrics properties (Ayalon and Gum, 2011; Palmore, 2001), interpretations about prevalence of ageism are questionable. This is the first study to systematically evaluate existing ageism scales. The 106 records included in this study assessed the psychometric properties of 11 scales. All scales evaluated in this review explicitly assess ageism. An explicit assessment of ageism enquires about people’s thoughts, feelings or behaviors towards older adults because of their age (Palmore, 2001), whereas an implicit assessment does not reveal that the focus of the assessment concerns age. Hence, there is no possible control over the responses given to implicit tests, which are thought to be free of social demand characteristics (Cherry et al., 2015; Greenwald et al., 2002).

Despite the number of scales available to explicitly measure ageism, only the Expectations Regarding Aging has adequate content validity, structural validity and internal consistency. Further studies are however required to get a clearer understanding of the cross-cultural validity, measurement error, criterion validity, and responsiveness of this scale. Moreover, the fact that this is an explicit scale that only assesses stereotypes precludes its use as a comprehensive ageism scale. The remaining ten scales included in this review need further psychometric evaluation and refinement. It is worth noting that two of the scales reviewed, the Attitudes to Aging Questionnaire and the Kogan’s Attitudes towards Older People Scale, which had adequate structural validity and internal consistency, may benefit from revisions to improve their content validity. Indeed, one important finding from this study is that the dimension(s) of ageism assessed by existing scales is not always clear. The concept ‘attitudes’ is often used to refer to several dimensions of ageism (e.g. stereotypes and prejudice) without clear indications of the intended meaning. Given the multi-dimensional nature of ageism, it is desired to have scales, which clearly address all three dimensions. The reference populations used across scales also varied with some scales including multiple references. Research has shown that older adults seem to distinguish between their own ageing and the ageing of others (Gething, 1994; Helmes and Pachana, 2016), so including multiple reference populations in a single scale may be beneficial.

In reviewing the findings, it is important to note the study’s limitations. Despite ongoing consultations with information specialists, the use of snowballing, and the conduct of specific bibliographic searches, it is possible that relevant articles were missed. In addition, the COSMIN guidelines do not yet offer an easy-to-use-format for data extraction and quality assessment. As a result, it is possible that errors were inadvertently made when extracting data, assessing properties and quality of the evidence. To overcome this limitation, two independent raters conducted the initial extraction and assessment, with at least a third rater checking and confirming the results.

## 5 Conclusions

This systematic review reveals a gap in the ageism field. Of all available ageism scales, only one met minimum requirements for psychometric validation but still failed to cover all dimensions of ageism. This review also revealed that there is a general lack of psychometric assessments of existing ageism scales with many having less than three independent studies as their evidence base. For those scales that do have assessments by three or more independent studies, evidence is often of low quality and /or provided on only a handful of psychometric properties. Moreover, studies often yield indeterminate or inconsistent results on the measurement properties assessed.

Without comprehensive and psychometrically valid ageism scales we may not be able to accurately assess the prevalence of ageism and evaluate if available strategies to reduce or prevent it work, which can result in poor investments and hinder global and national efforts to tackle ageism. This study signals a need to further study scales that evaluate explicit aspects, with a specific focus on those scales that measure the three dimensions of ageism. It also highlights the need to identify scales that evaluate implicit aspects of ageism. Even though it is possible that such scales exist, they have not been examined by independent research groups and are therefore still lacking psychometric support. Our findings also highlight the need for research in a more diverse group of countries, and the inclusion of a more diverse pool of participants. The development and validation of a new ageism scale that covers all dimensions of ageism, includes different reference targets (i.e. self and others), and accounts for both positive and negative ageism, and explicit and implicit manifestations of this phenomenon is desirable.

## Contributors

LA and VFN contributed equally to this work, and were involved in study conceptualization and design, data collection and screening, data extraction and synthesis, results interpretation and manuscript writing. PD, SM, JPB, GR, JS, MW contributed to data screening, data extraction and final drafting of the paper.

The Corresponding Author (VFN) had full access to all the data in the study and had final responsibility for the decision to submit for publication.

## Transparency declaration

The lead authors (LA, VFN) affirm that this manuscript is an honest, accurate, and transparent account of the study being reported; that no important aspects of the study have been omitted; and that any discrepancies from the study as planned (and, if relevant, registered) have been explained.

## Ethics approval

Not required for this study.

Declaration of Competing Interest

The authors have no conflict of interest to report.

## Funding

This research did not receive any specific grant from funding agencies in the public, commercial, or not-for-profit sectors.

## Figures and Tables

**Table:** 

**#**	**Search string**
**1**	(“Ageism”[MeSH] OR ageism[TiAb] OR agism[TiAb] OR ageist[TiAb] OR agist[TiAb] OR “age discrimination”[TiAb] OR “age prejudice”[TiAb] OR “age stereotype”[TiAb] OR “self perceptions of ageing”[TiAB] OR “self perceptions of aging”[TiAB] OR “age identity”[Tiab])
**2**	((“Aged”[Mesh] OR “Aged, 80 and over”[MeSH] OR “Frail Elderly”[MeSH] OR elder*[TiAb] OR “seniors”[TiAb] OR “older adult”[TiAb] OR “older person”[TiAb] OR “older adults”[TiAb] OR “older persons”[TiAb] OR “older peoples”[tiab] OR “older people”[TiAb] OR “aging”[MeSH] OR “ageing”[TiAb] OR “aging”[TiAb] OR “Old age”[Ti]) AND (“Social Exclusion”[Tiab] OR “social rejection”[TIab] OR “Social Acceptance”[TIab] OR “stereotyped behavior”[Mesh] OR “social perception”[Ti] OR “age identification”[ti] OR “self-perceptions”[tiAb] OR “Prejudice”[MeSH] OR “prejudice”[TiAb] OR stereotyp*[TiAb] OR “Stereotyping”[MeSH] OR “Social Discrimination”[Mesh] OR “Intergenerational Relations”[Mesh]))
**3**	(“Animals” NOT (“Animals”[Mesh] AND “Humans”[Mesh]))
**4**	Plants[Mesh]
**5**	Step 1 OR Step 2 NOT Step 3 NOT Step 4

## Appendix A “Ageism” Search terms for PubMed & Electronic databases

### PubMed search strategy

*Filter applied:* published after 1970

### Electronic databases searched

PubMed, PsychInfo, Ageline, CINAHL, EBSCO, EMBASE, Cochrane Database of Systematic Reviews, Global Index Medius, Campbell Collaboration, Prospero, Epistemonikos, DARE, Open Grey and Greynet.
