# Supplementary material for: A systematic review of existing ageism scales
Source: Ageing Res Rev. 2019 Sep;54:100919. doi: 10.1016/j.arr.2019.100919 (PMC6745621; doi:10.1016/j.arr.2019.100919)
Supplement: Supplementary file 1 [file mmc1.pdf]

Supplementary materials

Supplementary Appendix A
COSMIN definition of measurement properties

Measurement property	Definition
Content validity	The degree to which the content of a scale is an adequate reflection of the construct to be measured
Structural validity	The degree to which the scores of a scale are an adequate reflection of the dimensionality of the construct to be measured
Internal consistency	The degree of the interrelatedness among the items
Cross-cultural validity/Measurement invariance	The degree to which the performance of the items on a translated or culturally adapted scale are an adequate reflection of the performance of the items of the original version of the scale
Reliability	The proportion of the total variance in the measurements which is due to 'true' differences between participants
Measurement error	The systematic and random error of a participant's score that is not attributed to true changes in the construct to be measured
Criterion validity	The degree to which the scores of a scale are an adequate reflection of a 'gold standard' or longer version of a scale
Construct validity *(including both convergent and known groups validity)*	The degree to which the scores of a scale are consistent with hypothesis based on the assumption that the scale validly measures the construct to be measured
Responsiveness	The ability of a scale to detect change over time in the construct to be measured

Adapted with permission from (Mokkink et al., 2018)

Supplementary Appendix B
COSMIN Guidelines for the Assessment of Each Measurement Property in a Scale

	Rating*	Criteria
Content validity	+	A clear description of measurement aim, target population, concepts measured, item selection and target population and (investigators or experts) were involved in item selection.
	?	A clear description of above mentioned aspects is lacking OR only target population involved OR doubtful design or methods.
	-	No target population involved.
Structural validity	+	**CTT**<br>CFA: CFI or TLI or comparable measure > 0.95 OR RMSEA < 0.06 OR SRMR < 0.08†
		**IRT/Rasch**<br>No violation of unidimensionality‡: CFI or TLI or comparable measure > 0.95 OR RMSEA < 0.06 OR SRMR < 0.08<br>*AND*<br>no violation of local independence: residual correlations among the items after controlling for the dominant factor < 0.20 OR Q3's < 0.37<br>*AND*<br>no violation of monotonicity: adequate looking graphs OR item scalability > 0.30<br>*AND*<br>adequate model fit<br>IRT: $\chi^2 > 0.001$<br>Rasch: infit and outfit mean squares ≥ 0.5 and ≤ 1.5 OR Z-standardized values > −2 and < 2
	?	CTT: not all information for '+' reported<br>IRT/Rasch: model fit not reported
	−	Criteria for '+' not met
Internal consistency	+	At least low evidence§ for sufficient structural validity¶ AND Cronbach's alpha(s) ≥ 0.70 for each unidimensional scale or subscale‖
	?	Criteria for "At least low evidence§ for sufficient structural validity¶" not met
	−	At least low evidence§ for sufficient structural validity¶ AND Cronbach's alpha(s) < 0.70 for each unidimensional scale or subscale‖
Reliability	+	ICC or weighted Kappa ≥ 0.70
	?	ICC or weighted Kappa not reported
	−	ICC or weighted Kappa < 0.70

	Rating[*]	Criteria
Measurement error	+	SDC or LoA < MIC[¶]
	?	MIC not defined
	−	SDC or LoA > MIC[¶]
Hypotheses testing for construct validity	+	The result is in accordance with the hypothesis[**]
	?	No hypothesis defined (by the review team)[††]
	−	The result is not in accordance with the hypothesis[**]
Cross-cultural validity\measurement invariance	+	No important differences found between group factors (such as age, gender, language) in multiple group factor analysis OR no important DIF for group factors (McFadden's $R^2 < 0.02$)
	?	No multiple group factor analysis OR DIF analysis performed
	−	Important differences between group factors OR DIF was found
Criterion validity	+	Correlation with gold standard ≥ 0.70 OR AUC ≥ 0.70
	?	Not all information for '+' reported
	−	Correlation with gold standard < 0.70 OR AUC < 0.70
Responsiveness	+	The result is in accordance with the hypothesis[**] OR AUC ≥ 0.70
	?	No hypothesis defined (by the review team)[††]
	−	The result is not in accordance with the hypothesis[**] OR AUC < 0.70

Reprinted with permission from (Mokkink et al., 2018)

AUC: Area under the curve, CFA: Confirmatory factor analysis, CFI: Comparative fit index, CTT: Classical test theory, DIF: Differential item functioning, ICC: Intraclass correlation coefficient, IRT: Item response theory, LoA: Limits of agreement, MIC: Minimal important change, RMSEA: Root Mean Square Error of Approximation, SEM: Standard Error of Measurement, SDC: Smallest detectable change, SRMR: Standardized Root Mean Residuals, TLI: Tucker-Lewis Index

[*] "+" = sufficient, "–" = insufficient, "?" = indeterminate

[†] To rate the quality of the summary score, the factor structures should be equal across studies

[‡] Unidimensionality refers to a factor analysis per subscale, while structural validity refers to a factor analysis of a (multidimensional) patient-reported outcome measure

[§] As defined by grading the evidence according to the GRADE approach

[¶] This evidence may come from different studies

[‖] The criteria 'Cronbach alpha < 0.95' was deleted, as this is relevant in the development phase of a PROM and not when evaluating an existing PROM.

[**] The results of all studies should be taken together, and it should then be decided if 75% of the results are in accordance with the hypotheses

[††] We relied on hypotheses specified by the authors, rather than the review team

Supplementary Appendix C
Aggregated Grading of the Quality of Evidence for each Measurement Property per Scale

Quality of evidence	Lower if
**High**<br>**Moderate**<br>**Low**<br>**Very low**	**Risk of Bias**<br>**-1 Serious** (multiple studies of doubtful quality or only one study of adequate quality)<br>**-2 Very Serious** (multiple studies of inadequate quality or only one study of doubtful quality)<br>**-3 Extremely Serious** (there is only one study of inadequate quality)<br><br>**Inconsistency**<br>**-1 Serious** (inconsistent results across studies OR inconsistent number of items and sub-scales proposed across studies)<br>**-2 Very Serious** (inconsistent results across studies or inconsistent number of items AND sub-scales proposed across studies)<br><br>**Imprecision**<br>**-1 total n=50/100**<br>**-2 total n <50**

Adapted with permission from (Mokkink et al., 2018)

Supplementary Table 1
Study Characteristics

Author	Specific questionnaire	Age range (M, SD)	Gender (% female)	Population type	Country	Language	N (measurement property tested)	Sampling method	Response rate
**Aging Perceptions Questionnaire (APQ)**									
(Barker et al., 2007)	APQ	(M=74.1, SD=6.8)	57%	Community dwelling adults	Ireland	English?	2033 (Structural validity, internal consistency)	Random	68%
(Barker et al., 2007)	APQ	Not reported	Not reported	Community dwelling adults above 65 years	Ireland	English?	143 (Reliability, construct validity - convergent)	Not reported	Not reported
(Barker et al., 2007)	APQ	Not reported	Not reported	Expert clinicians and researchers in aging	Ireland	English?	16 (Content validity - scale development)	Convenient	Not reported
(Barker et al., 2007)	APQ	Not reported	Not reported	Community dwelling adults above 65 years	Ireland	English?	129 (Content validity? - pilot test psychometric properties)	Convenient?	Not reported
(Ingrand et al., 2012)	APQ	Subgroup 1= 65 to 106 (M=77.4, SD=8.2). Subgroup 2= 54 to 64 (M=59.8, SD=2.8)	56% (Subgroup 1=58%; Subgroup 2=53%)	All inhabitants of Neuville de Poitou aged 55 years or more	France	French	656, including Subgroup 1 with participants aged 65+ (394), and Subgroup 2 with participants aged 55 to 65 (262). (Structural validity, internal consistency, cross-cultural validity/ measurement invariance, construct validity - known groups)	Total populati on, sampling all inhabitants 55+	Subgroup 1=97%; Subgroup 2=98%
(Sexton et al., 2014)	Brief Ageing Perceptions Questionnaire (B-APQ)	56 to 70	54%	Community dwelling individuals aged 50+	Ireland	English?	6718 (Structural validity, internal consistency, criterion validity, construct validity - convergent)	Representativ e 50+	62%?
(Slotman et al., 2015)	Aging Perceptions Questionnaire – Short (APQ-S)	70 to 99 (M=78.59, SD=6.17)	57%	Community dwelling older people in different neighborhoods in Rotterdam	Netherlands	Dutch	1280 (Structural validity, internal consistency, cross-cultural validity/measurement invariance, criterion validity, construct validity - convergent & known groups)	Random	46%
(Chen et al., 2016)	APQ	(M=71.80, SD=6.48)	53%	Not reported	China	Chinese	94 (Content validity? - pilot test) of which 30 (reliability)	Convenient	94% (pilot study); 100% (reliability)
(Chen et al., 2016)	APQ	Not reported	Not reported	Gerontology expert, linguistics expert, psychologists, nursing psychological experts	China	Chinese	7 (Content validity - adaptation of questionnaire to Chinese context)	Convenient?	Not reported
(Chen et al., 2016)	APQ	Not reported	Not reported	Older adults 65+	China	Chinese	20 (Content validity - pilot test clarity and intelligibility)	Convenient 65+	Not reported

Author	Specific questionnaire	Age range (M, SD)	Gender (% female)	Population type	Country	Language	N (measurement property tested)	Sampling method	Response rate
(Chen et al., 2016)	APQ	EFA sample (M=72.38, SD=6.536); CFA sample (M=73.75, SD=6.767)	EFA sample= 52%; CFA sample= 53%	Not reported	China	Chinese	758 including two sub-samples to conduct exploratory factor analysis (EFA) and confirmatory factor analysis (CFA) for the assessment of structural validity (Structural validity, internal consistency)	Convenient	84%
(Moghadam et al., 2016)	B-APQ	Not reported	Not reported	Clinical psychologist, psychiatrist, assistant professors in nursing, assistant professors experienced in questionnaire design	Iran	Persian	8 (Content validity)	Convenient?	Not reported
(Moghadam et al., 2016)	B-APQ	Not reported	Not reported	Older adults	Iran	Persian	10 (Content validity - pilot test understandability)	Convenient?	Not reported
(Moghadam et al., 2016)	B-APQ	60 to 75 (72.8% of sample); 76 to 90 (24.4% of sample); 90+ years (2.8% of sample)	49%	Community dwelling older adults >60 years living in Gonabald, Iran and recruited from health centres	Iran	Persian	400 (structural validity, internal consistency) of which 20 (reliability)	Two-stage random cluster sampling method	95%
(Slotman et al., 2017)	APQ-S	65 to 99 (M=72.82, SD=5.21)	45%	Community-dwelling Turkish migrants who resided in Rotterdam	Netherlands	Turkish	428 (Structural validity, internal consistency, cross-cultural validity/measurement invariance, construct validity - convergent & known groups)	Unclear	20%
**Aging Semantic Differential (ASD)**									
(Rosencranz and McNevin, 1969)	ASD	17 to 21	Not reported	University students	USA	English	200 (Content validity)	Not reported	Not reported
(Rosencranz and McNevin, 1969)	ASD	Not reported	Not reported	University students	USA	English	287 (Structural validity, construct validity - known groups)	Not reported	Not reported
(Underwood et al., 1985)	ASD	Not reported	Approx. 99%	University students	USA	English?	184 (Structural validity)	Convenient	Not reported
(Gekoski et al., 1991)	ASD	Not reported	Not reported	University students	Canada	English?	100 (Structural validity)	Convenient	Not reported
(O'Hanlon et al., 1993)	*ASD*	*Young age group=20 to 30 (M=20.3, SD=2.87); Middle-aged group=31 to 53*	*64%*	*Adults of all ages attending four different colleges in New Orleans*	*USA*	*English?*	*387, including three separate age groups: young (279), middle-aged (50), old (58).(Construct validity - convergent & known groups)*	*Convenient*	*Not reported*

**Author**	**Specific questionnaire**	**Age range** (M, SD)	**Gender** (% female)	**Population type**	**Country**	**Language**	**N** (measurement property tested)	**Sampling method**	**Response rate**
		(M=38.2, SD=5.33); Old age group=64 to 85 (M=70.03, SD=5.33)							
(Intrieri et al., 1995)	ASD	(M=26.2, SD=3.76)	24%	Third year medical students enrolled in a geriatric training programme	USA	English?	100 (Structural validity, internal consistency)	Convenient	76%
(Villar Posada, 1997)	Spanish version of the ASD (Diferencial Semántico del envejecimiento)	20 to 56 (M=31.1)	53%	People living in the metropolitan area of Barcelona	Spain	Spanish	82 (Content validity - pilot test)	Purposeful sampling	Not reported
(Villar Posada, 1997)	Spanish version of the ASD (Diferencial Semántico del envejecimiento)	15-no upper age limit	Approx. 50%	Community dwellers living in the metropolitan area of Barcelona	Spain	Spanish	166 (Structural validity, internal consistency, construct validity - convergent)	Purposeful sampling considering gender and age	Not reported
(Polizzi and Millikin, 2002)	The Refined Aging Semantic Differential (R-ASD)	18 to 23 years	84%	Undergraduate students enrolled in education and psychology courses at a southern liberal arts university	USA	English?	142 including three sub-samples, each responding to a differently worded target (Construct validity - known groups)	Convenient	100%
(Polizzi, 2003)	R-ASD	Not reported	Not reported	Doctoral level experts in the areas of adult development and aging, psycholinguistics, and cultural diversity.	USA	English?	9+ people (Content validity)	Convenient?	Not reported
(Polizzi, 2003)	R-ASD	17 to 22	55%	University students	USA	English?	300 (Structural validity, internal consistency)	Convenient	Not reported
(Polizzi, 2003)	R-ASD	17 to 22	60%	University students	USA	English?	50 (Reliability)	Convenient	Not reported
(Stewart et al., 2007)	ASD	Not reported	Not reported	Medical students (2 cohorts as quasi control, 2 cohorts as treatment)	USA	English? Not specifically reported	Quasi control Group1 test time 1=53; Quasi-control Group 1 test time 2= 73; Quasi control Group2 test time 1=55; Quasi-control Group 2 test time 2= 63; Intervention Group 1 test time 1=70; Intervention Group 1 test time 2=68; Intervention Group 1 test time 3=65; Intervention Group 2 test time 1=71; Intervention Group 2 test time 2=68. (Internal consistency, responsiveness)	Not reported	Not reported

Author	Specific questionnaire	Age range (M, SD)	Gender (% female)	Population type	Country	Language	N (measurement property tested)	Sampling method	Response rate
(Iwasaki and Jones, 2008)	R-ASD	(M=21.6, SD=3.8)	54%	Undergraduate college students enrolled in counseling psychology	USA	English?	512 (Internal consistency, Construct validity - convergent)	Convenient	Not reported
(Iwasaki and Jones, 2008)	R-ASD	(M=21.2, SD=4.57)	60%	College students-various courses	USA	English?	733 (Structural validity)	Convenient	93%
(Boudjemad and Gana, 2009)	*ASD*	*(M=23.17, SD=6.25)*	*Not reported*	*Bilingual students*	*France?*	*French*	*32 (Internal consistency, cross- cultural validity/measurement invariance)*	*Convenient?*	*Not reported*
(Gluth et al., 2010)	ASD	Younger group=18 to 31 (M=24.8, SD=3.1); Older group=68 to 81(M=73.4, SD=3.1)	Younger group=56%; Older group=57%	Younger and older German participants recruited through posters or handouts posted and distributed at grocery stores and swimming pools, and ads in newspapers	Germany	German	294 including a younger age sub-group (151) and an older age sub-group (143).(Structural validity, internal consistency, cross-cultural validity/measurement invariance, construct validity - convergent & known groups)	Convenient	Not reported
(Gonzales et al., 2010)	R-ASD	Not reported	Not reported	Social gerontologist and graduate students in gerontology	USA	English?	3 (Content validity)	Not reported	Not reported
(Gonzales et al., 2010)	R-ASD	21 to 37	Not reported	Medical students from eight universities across the country	USA	English?	112 (Content validity)	Not reported	42%
(Gonzales et al., 2010)	R-ASD	21 to 37 (M=24, SD=2.4)	61%	Medical students from eight universities across the country	USA	English?	199 (Structural validity, internal consistency)	Convenient?	Not reported
(Carlson, 2015)	R-ASD	Not reported	Not reported	Graduate students	USA	English?	19 (Content validity)	Not reported	Not reported
(Carlson, 2015)	R-ASD	Sub-group A= 17 to 25 (89% of sub-group); Sub-group B= 17 to 25(85% of sub-group)	Sub-group A=66%; Sub-group B= 60%	University students	USA	English?	Two subgroups: sub-group A tested 5-point Likert scale (258) and Sub-group B tested 11-point Likert scale (233). (Structural validity, internal consistency, construct validity -convergent & known groups)	Convenient	Sub-group A: 94%; Sub-group B Liker 90%
(Gonzales et al., 2017)	ASD	18 to 36 (M=21.6, SD=2.2)	69%	College students (undergraduate and graduate students) at same university in Shangai	China	Mandarin	380 (Structural validity, internal consistency, cross-cultural) validity/measurement invariance)	Convenient	100%
**Anxiety about Aging Scale (AAS)**									
(Lasher, 1987)	AAS	Not reported	Not reported	Psychology graduates	USA	English?	3 (Content validity)	Not reported	Not reported

Author	Specific questionnaire	Age range (M, SD)	Gender (% female)	Population type	Country	Language	N (measurement property tested)	Sampling method	Response rate
(Lasher, 1987)	AAS	Not reported for whole sample	35%	Students in undergraduate psychology courses and from Foster Grandparent Program	USA	English?	312 including seven age groups: <25 (n=46), 25 to 34 (n=47), 35 to 44 (n=46), 45 to 54 (n=56), and 55 to 64 (n=39). (Structural validity, internal consistency, construct validity - convergent & known groups)	Convenient & snowballing	84.8%
(Watkins et al., 1998)	AAS	65 to 74 (49%); 75 to 87 (51%)	71.50%	Community dwelling older adults	Australia	English?	123 (Structural validity, internal consistency, cross-cultural validity/measurement invariance, construct validity - known groups)	Convenient	96%
(Rivera-Ledesma et al., 2007)	AAS	(M=63.8, SD=8.7)	55%	Older adults selected based on their availability in a health clinic in Mexico City. It was subjects who attended the clinic for a routine checkup or a follow up visit.	Mexico	Spanish	234 (Structural validity, internal consistency, construct validity - convergent)	Convenient	Not reported
(Rivera-Ledesma et al., 2007)	Revised Anxiety about Ageing Scale (R-AAS)	(M=63, SD=8.2)	54%	Older adults selected based on their availability in a health clinic in Mexico City. It was subjects who attended the clinic for a routine check up or a follow up visit.	Mexico	Spanish	151 (Structural validity, internal consistency, construct validity - convergent)	Convenient	Not reported
(Gao, 2012)	AAS	Students= 19 to 32 (M=20.6), Older adults=50 to 84 (M=62.9)	62%	Psychology students from two universities, and older adults-volunteers from two community colleges	China (Taiwan)	Chinese	334, including students (176) and older adults (158). (Structural validity, internal consistency, cross-cultural validity/measurement invariance, construct validity - known groups)	Convenient	Not reported
(Koukouli et al., 2013)	AAS	Not reported	Health and social care professionals (68%); Senior undergraduate students (85%); Community residents (50%)	Health and social care professionals; senior undergraduate students enrolled in the Health and Social Welfare School of TEI of Crete; community workers living in Crete	Greece	Greek	320, including three sub-samples: health and social care professionals (147), senior undergraduate students (74) and community residents (99). (Structural validity, internal consistency, construct validity - known groups)	Professionals and students: Convenient? Sample of community residents: snowballing	Health and social care professionals (>80%); Students (86%); Community residents (50%)
(Sargent-Cox et al., 2014)	AAS	(M=57.3, SD= 13.66)	42%	Residents of Australian Capital Territory	Australia	English?	783 (Structural validity, internal consistency, cross-cultural validity/measurement invariance)	Random	23.5% respondents; of these 84.3% complete cases

Author	Specific questionnaire	Age range (M, SD)	Gender (% female)	Population type	Country	Language	N (measurement property tested)	Sampling method	Response rate
**Attitudes to Ageing Questionnaire (AAQ)**									
(Laidlaw et al., 2007)	AAQ	60+	Approx. 66%	Older adults	UK (Scotland)	English?	35+ (Content validity)	Convenient	Not reported
(Laidlaw et al., 2007)	AAQ	>60 in the older adults' groups	50% in the older adults' groups	15 centers*(4 groups of older adults, 1 group of carers, 1 group of health care professionals)	Several countries	English?	About 6 focus groups in 15 centers (Content validity)	Not reported	Not reported
(Laidlaw et al., 2007)	AAQ	60 to 99	60%	WHOQOL-OLD centres: older adults	Several countries	Not reported	1,356 (Structural validity, cross-cultural validity/measurement invariance)	Convenient	Not reported
(Laidlaw et al., 2007)	AAQ	60 to 100	59%	WHOQOL-OLD centres: older adults	Several countries	Not reported	5,566 (Structural validity, internal consistency, cross-cultural validity/measurement invariance)	Convenient	Not reported (83% complete cases)
(Chachamovich et al., 2008)	AAQ	60 to 69	59%	Not reported	Brazil	Portuguese?	143 (Content validity)	Convenient	Not reported
(Chachamovich et al., 2008)	AAQ	60 to 69 (41%); 70 to 79 (36%); 80 to 89 (23%)	64%	Older adults recruited from a university hospital, community houses and nursing homes, elderly community groups, and their own homes	Brazil	Portuguese?	424 (Structural validity, internal consistency, cross-cultural validity/measurement invariance, construct validity - convergent)	Convenient	Not reported
(Kalfoss et al., 2010)	AAQ	60 to 95 (M=72.32, SD=7.8)	54%	Community dwellers, 60+ years old	Canada	English	202 (Structural validity, internal consistency, construct validity - convergent & known groups)	Convenient	48%
(Kalfoss et al., 2010)	AAQ	60 to 91 (M=77.62, SD=7.2)	57%	Community dwellers and those receiving health care services	Norway	Norwegian	490 (Structural validity, internal consistency, construct validity - convergent & known groups)	A cohort of randomly selected stratified older adults from 20 communities; and another cohort of randomly selected older adults receiving health care services	Approx. 53%
(Lucas-Carrasco et al., 2013)	AAQ	(M= 71.1, SD=7.1)	60%	Community dwellers 60+ recruited from community centres, primary care centres,	Spain	Spanish	242 (Structural validity, internal consistency, construct validity - convergent & known groups)	Convenient	Not reported

Author	Specific questionnaire	Age range (M, SD)	Gender (% female)	Population type	Country	Language	N (measurement property tested)	Sampling method	Response rate
				and family associations for the mentally ill and dementia					
(Shenkin et al., 2014)	AAQ	(M=74.0, SD= 0.28)	48%	Community dwellers (the Lothian Birth Cohort 1936)	UK (Scotland)	English	802 (Structural validity, internal consistency, construct validity - convergent)	The Lothian Birth Cohort 1936 - participants of 2nd wave	95.3%
(Brown et al., 2015)	AAQ	40 to 60 (M = 52.1, SD = 5.49); and 60 to 98 (M = 71.3, SD = 7.69)	100%	Community dwellers aged between 18 and 98 recruited from the Australian electoral roll	Australia?	English?	776, including two sub-groups of midlife women (571) and older women (259). (Structural validity, cross-cultural validity/ measurement invariance, construct validity - convergent)	Random	Older group (34.9%); younger group (35.7%)
(Helmes and Pachana, 2016)	*AAQ*	*Canadian sample: 50 to 94 (M=70.3, SD=8.88). Australian sample: 50 to 90 (not reported)*	*Canadian sample: 69%. Australian sample: 74%*	*Both Canadian and Australian samples: Community dwelling older adults*	*Canada & Australia*	*English?*	*Canadian sample: 172 Australian sample: 195 (Internal consistency, construct validity - convergent)*	*Convenient*	*Canadian sample: 56%; Australian sample: 95%*
(Marquet et al., 2016)	AAQ	(M=73.84, SD=8.66)	66%	French speaking older people living in Belgium	Belgium	French	238 (Structural validity, internal consistency, reliability, construct validity - convergent & known groups)	Convenient	Not reported
(Rejeh et al., 2017)	AAQ	Not reported	Not reported	Older adults	Iran	Farsi	15 (Content validity)	Not reported	Not reported
(Rejeh et al., 2017)	AAQ	Not reported	Not reported	Specialists (1 geriatrician, 1 psychologist, 3 associate professors in nursing, 1 questionnaire development expert)	Iran	Farsi	6 (Content validity)	Not reported	Not reported
(Rejeh et al., 2017)	AAQ	(M=67.39, SD=6.14)	54%	Community dwellers, members of citizen clubs	Iran	Farsi	400 (Structural validity, internal consistency, construct validity - known groups). And a sub-sample of 80 (reliability).	Random sampling within clubs in 4 regions of Tehran	Not reported
**Expectations Regarding Aging (ERA)**									
(Sarkisian et al., 2001)	ERA	65 to 91 (M=78) in the older adults group. 30 to 51 (M=37) in the	34% for older adult group. 54% for physician group	Older adults aged 65+ attending community-based senior centers in which most clients spoke English. Also full time primary care physicians working in the	USA	English	49 including 38 older adults and 11 primary care clinicians. (Content validity - PROM development)	Convenient	80% older adults

Author	Specific questionnaire	Age range (M, SD)	Gender (% female)	Population type	Country	Language	N (measurement property tested)	Sampling method	Response rate
		primary care clinicians group		division of geriatrics and general internal medicine					
(Sarkisian et al., 2001)	ERA	Not reported	Focus groups of older adults 100%	Community based older adults from different socioeconomic and demographic backgrounds	USA	English	6 older adults for individual interviews, and 12 older women for a focus group discussion. (Content validity - PROM development)	Convenient?	Not reported
(Sarkisian et al., 2002)	ERA-38	M=76	54%	Community residing older adults (>65 years) cared for by primary care physicians	USA	English	429 (Content validity, internal consistency, construct validity - convergent & known groups)	Random sample from each physician, but physicians were not randomly selected	73%
(Sarkisian et al., 2002)	ERA	M=78 (older adults in focus group discussions)	Not reported	Older adults and physicians	USA	English	38 older adults and an unknown number of physicians for focus group discussions, 6 interviewees (Content validity - PROM development)	Not reported	Not reported
(Sarkisian et al., 2002)	ERA	Not reported	Not reported	Older adults in 2 senior centers	USA	English	58 (Content validity - pilot)	Not reported	Not reported
(Sarkisian et al., 2005)	ERA-38 & ERA-12	(M=76, SD=6.9)	54%	Community residing older adults (>65 years) cared by primary care physicians	USA	English	429 (Content validity, structural validity, internal consistency, criterion validity, construct validity – convergent & known groups)	Random sample from each physician	73%
(Sarkisian et al., 2005)	ERA-38 & ERA-12	(M=77.5, SD=6.5)	77%	Older adults aged 65 years and older at 14 senior centers and able to speak either English or Spanish	USA	English and Spanish	636 (Structural validity, internal consistency, cross cultural validity/measurement invariance, construct validity-convergent & known groups). 20% of these (reliability)	Convenient?	85%
(Joshi et al., 2010)	ERA-12	41 to 62 (41 to 51=43.4%, 52 to 62=63.6%)	64%	Middle-aged individuals able to read and understand English, seeking treatment in policlinics, and including 3 main ethnic groups (i.e. Chinese, Malay and Indian) in Singapore	Singapore	English	981 (Structural validity, internal consistency, construct validity-convergent & known groups)	Not reported	48.3%
(Beser et al., 2012)	ERA-12	Not reported	Not reported	1 family physician, 8 faculty members nursing school, 1 clinical nurse	Turkey	Turkish	10 (Content validity)	Convenient?	Not reported
(Beser et al., 2012)	ERA-12	Not reported	Not reported	Older adults 65+	Turkey	Turkish	10 (Content validity - pilot)	Convenient?	Not reported

Author	Specific questionnaire	Age range (M, SD)	Gender (% female)	Population type	Country	Language	N (measurement property tested)	Sampling method	Response rate
(Beser et al., 2012)	ERA-12	65 to 86 (M=71.68, SD=5.37)	54%	People aged 65 and above, living in Izmir, and with no hearing or speaking problems	Turkey	Turkish	120 (Structural validity, internal consistency)	Convenient?	Not reported
(Sparks et al., 2013)	ERA-38, with two underlying scales: Aging Self Expectations-ASE, and General Aging Expectations-GAE	45 to 74 (M= 59.46, SD = 8.18)	60%	People attending various adult physical recreational and sport programs as well as members of non-physically active community organizations, and staff at secondary schools	Canada	English?	167 (Structural validity, internal consistency, construct validity - known groups)	Convenient?	Not reported
**Facts on Aging Quiz (FAQ)**									
(Palmore, 1977)	FAQ	Not reported	Not reported	Graduate and undergraduate students and faculty from University campus	US	English?	142 (Construct validity - known groups)	Convenient?	Not reported
(Klemmack, 1978)	FAQ	(M=40.2, SD= 15.3)	51%	Community dwellers	US	English?	202 (Structural validity)	Stratified random	Not reported
(Holtzman and Beck, 1979)	FAQ	(M= 28.3)	46%	University students, health workers	US	English?	527 (Construct validity - convergent & known-groups)	Convenient	Not reported
(Miller and Dodder, 1980)	FAQ *& Miller-Dodder Revision (MDR)*	*(M=18.5)*	*64%*	*College students*	*US*	*English?*	*430 with a sub-sample of 207 responding to FAQ and a sub-sample of 223 responding to the Miller-Dodder revised scale (Construct validity - convergent)*	*Convenient*	*Not reported*
(Laner, 1981)	FAQ	No details (50% reported to be close to 20; next largest age group reported to be close to 30)	66% (intervention group); No details reported (control group)	Social gerontology university students (intervention group) and sociology university students not engaged in gerontological topics (control group)	US	English?	Intervention group=46 (first take of questionnaire before commencement gerontology course) and 37 (second take of questionnaire at the end of gerontology course). Control group= 43 (first take of questionnaire) and 38 (second take of questionnaire). (Construct validity - known groups, responsiveness)	Not reported	84% for second wave in intervention group, and 88% for second wave in control group
(Laner, 1981)	FAQ	No details (majority close to age 20; next largest age group reported to be close to 30)	80% (intervention group); 50% (control group)	Social gerontology university students (intervention group) and sociology university students not engaged in gerontological topics (control group)	US	English?	Intervention group=30 (first take of questionnaire before commencement gerontology course) and 24 (second take of questionnaire at the end of gerontology course). Control group= 42 (first take of questionnaire) and 31 (second take of questionnaire). (Construct validity - known groups, responsiveness)	Not reported	80% for second wave

**Author**	**Specific questionnaire**	**Age range** (M, SD)	**Gender** (% female)	**Population type**	**Country**	**Language**	**N** (measurement property tested)	**Sampling method**	**Response rate**
(Luszcz, 1982)	FAQ	First year students - 17 to 49 (M=22); Third year students - 19 to 55 (M= 27)	52% (first year students); 67% (third year students)	First year and third year university students	Australia	English?	218 of which 166 (first year university students) and 52 (third year students). (Content validity only with third year university students, construct validity - known groups including both sub-samples)	Convenient	Not reported
(Romeis and Sussman, 1982)	FAQ	US - 20 to 34 (18% of sample), 35 to 49 (40% of sample), 50+ (42% of sample). Japan - 20 to 35 years (38% of sample), 35 to 49 (62% of sample)	62% (US) 52% (Japan)	US - married persons, aged 20+ from North Carolina. Japan - married persons aged 20-49 living in Tokyo	US, Japan	English, Japanese?	206 (US), 591 (Japan) (Cross-cultural validity/measurement invariance)	Random	Not reported
(Matthews et al., 1984)	FAQ	Not reported	Not reported	Undergraduates and faculty from university, health professionals	Canada	English?	583 including 5 groups: first year students (258), third year students (83), third year students who had completed a course on gerontology (64), faculty (27), public health nurses (153). Samples selected to provide a comparison between students at varying levels of learning with respect to gerontology, and between students and two professional groupings, one academic and the other not. (Cross-cultural validity/measurement invariance, construct validity -known groups)	Convenient	Not reported
(Miller and Dodder, 1984)	FAQ & *MDR*	*M=18.5*	*64%*	*Students*	*US*	*English?*	*430 of which 207 completed FAQ and 223 MDR.(Construct validity - convergent & known groups)*	*Convenient*	*Not reported*
(Courtenay and Weidemann, 1985)	*FAQ 1 and 2*	*Not reported*	*69%*	*Students enrolled in undergraduate educational psychology*	*US*	*English?*	*141, subdivided into four subgroups (of 34, 31, 39 and 47 participants) that were given one of two versions of the quiz, and one of two response options. (Internal consistency, construct validity - known groups)*	*Convenient*	*Not reported*
(Dail and Johnson, 1983, 1986)	FAQ	Intervention group=18 to 44 (M=22.9). Control group	Intervention group=93%. Control group=91%	Undergraduates in a Midwestern university. Intervention group - enrolled in a human development course, which covered old	US	English?	Intervention group=61, control group=61. (Construct validity - known groups, responsiveness)	Convenient	Not reported

Author	Specific questionnaire	Age range (M, SD)	Gender (% female)	Population type	Country	Language	N (measurement property tested)	Sampling method	Response rate
		=18 to 39 (M=20.7)		age; Control group - enrolled in child-development					
(McCutcheon, 1986)	*Psychological Facts on Aging Quiz (PFA)*	*Not reported*	*51%*	*Students in psychology class*	*Canada*	*English?*	*121(Construct validity - known groups)*	*Convenient*	*Not reported*
(Donnelly et al., 1987)	*FAQ & Dye and Saddenrath Aging Quiz*	*Not reported*	*Not reported*	*Second year medical students, fourth year medical students, physicians in second or third year medical residency*	*US*	*English*	*Three separate groups including second year medical students (95); fourth year medical students (12); and physicians (33). (Construct validity - convergent & known groups, responsiveness)*	*Convenient*	*15%(2nd year students), 80% (4th year students), 89% (physicians)*
(Norris et al., 1987)	FAQ	Not reported	Not reported	First and third-year Students and faculty of university, and public health nurses	Canada	English?	Three separate groups including first-year students (258); third-year students (83); third-year students completing an introduction to gerontology (64); faculty (27); and public health nurses (153). Samples were selected to provide a comparison between students with varying levels of knowledge about gerontology and between these students and two professional groupings, one academic and the other not. (Structural validity, internal consistency, construct validity -known groups)	Convenient	Not reported
(Kline et al., 1990)	*FAQ &Knowledge of Aging and the Elderly (KAE)*	*19 to 23*	*50%*	*College-aged adults*	*Canada*	*English?*	*56 with half of the sample randomly assigned to respond to FAQ and half to KAE. (Construct validity - known groups)*	*Not reported*	*Not reported*
(Kline et al., 1990)	*FAQ & KAE*	*Not reported*	*Not reported*	*Students taking different university courses*	*Canada*	*English?*	*95 in total with some students taking a psychology of ageing course (19), others a course on life-span personality (43), and yet others taking a non-gerontological social science course (33).(Construct validity - known groups)*	*Convenient*	*Not reported*
(Kline and Kline, 1991a)	FAQ	25 to 66 (M= 45.3, SD=10.5)	88%	University students enrolled in gerontology program night classes	Canada?	English?	42 (Construct validity - known groups)	Convenient	Not reported
(Kline and Kline, 1991b)	*FAQ & KAE*	*17 to 45 (M=19.85, SD=4.60)*	*63%*	*Undergraduate students*	*Canada*	*English?*	*230 (Construct validity - convergent)*	*Convenient?*	*98%?*
(O'Hanlon et al., 1993)	*FAQ & KAE*	*Young age group=20 to 30 (M=20.3, SD=2.87).*	*64%*	*Adults of all ages attending four colleges in New Orleans*	*USA*	*English?*	*387, divided into three age groups including a younger age group (279), a middle-aged group (50), and an older age group (58)*	*Convenient*	*Not reported*

Author	Specific questionnaire	Age range (M, SD)	Gender (% female)	Population type	Country	Language	N (measurement property tested)	Sampling method	Response rate
		Middle-aged group=31 to 53 (M=38.2, SD=5.33). Old age group= 64 to 85 (M=70.03, SD=5.33)					*(Construct validity - convergent & known groups)*		
(Lusk et al., 1995)	*FAQ 1 & 2*	*(M=19.7, SD=3.1)*	*Not reported*	*Freshman nursing students*	*USA*	*English?*	*63 (Structural validity, internal consistency, construct validity - convergent & known groups)*	*Convenient*	*Not reported*
(Harris and Changas, 1994)	*FAQ 2*	*Not reported*	*Not reported*	*University students (sociology)*	*USA*	*English?*	*375 with 195 being given a multiple choice quiz format and 180 the standard true/false response format for the quiz (Internal consistency, construct validity – convergent, known groups)*	*Convenient?*	*Not reported*
(Harris et al., 1996)	*FAQ 1*	*Not reported*	*Not reported*	*University students (introduction to sociology)*	*USA*	*English?*	*230 True/False version, 271 Multiple choice version (internal consistency, measurement error, construct validity-convergent, known groups)*	*Convenient?*	*Not reported*
(Kramer et al., 2001)	*FAQ (true/false format -TF, and multiple-choice format - MC)*	*Not reported*	*Not reported*	*Health professionals, including trainees*	*USA*	*English?*	*69 physicians completed the true/false response format quiz, and 32 second-year medical students completed the multiple-choice format. (Construct validity - convergent). Sub-sample of 30 from the 69 physicians (content validity)*	*Convenient*	*Not reported*
(Pennington, 2001)	*FAQ 1 (TF, Don't Know format - DK)*	*Not reported*	*Not reported*	*An economist and a historian*	*New Zealand*	*English?*	*2 (Content validity - adaptation of quiz to New Zealand)*	*Convenient?*	*Not reported*
(Pennington, 2001)	*FAQ 1 (TF, DK)*	*Not reported*	*Not reported*	*Undergraduate, first year and third year university students*	*New Zealand*	*English?*	*218, including two sub-samples: participants who completed the true/false version (104) and participants who completed the 'don't know' version (109). (Internal consistency for each sub-sample, cross-cultural validity/measurement invariance, construct validity - convergent & known groups)*	*Convenient*	*Not reported*
(Obiekwe, 2001)	*FAQ I & II (TF & MC)*	*Not reported*	*Not reported*	*Freshmen students*	*USA*	*English?*	*995, including four sub-samples: participants who completed the FAQ II TF (270), participants who completed the FAQ II MC (230), participants who completed the FAQ I TF (255) and participants who completed the FAQ I MC (240). (Structural validity, internal consistency for each*	*Convenient*	*Not reported*

**Author**	**Specific questionnaire**	**Age range** (M, SD)	**Gender** (% female)	**Population type**	**Country**	**Language**	**N** (measurement property tested)	**Sampling method**	**Response rate**
							subsample, and construct validity - convergent across quiz formats)		
(Seufert and Carrozza, 2002)	*FAQ 1 & 2*	*Not reported*	*Not reported*	*Creator of original questionnaire, Palmore*	*USA*	*English?*	*1 (content validity)*	*Not reported*	*Not reported*
(Seufert and Carrozza, 2002)	*FAQ 1 & 2*	*Not reported*	*Not reported*	*Registered nurses*	*USA*	*English?*	*526 (Internal consistency, construct validity - convergent & known groups)*	*Random*	*60%*
(Cowan et al., 2004)	Revised Facts on Aging Quiz (R-FAQ)	Not reported	Not reported	Support workers employed in UK care homes for older people	UK	English?	155 (Internal consistency)	Convenient	75%
(Runkawatt, 2007)	*FAQ*	*Not reported*	*Not reported*	*Experts in geriatrics*	*Thailand*	*Thai*	*3 (Content validity)*	*Convenient*	*Not reported*
(Runkawatt, 2007)	*FAQ*	*Not reported*	*Not reported*	*Students from public high schools (12th graders)*	*Thailand*	*Thai*	*10 (Content validity - pilot test readability and understandability)*	*Convenient*	*Not reported*
(Runkawatt, 2007)	*FAQ*	*16 to 19 (M=17.2, SD=0.52)*	*60%*	*Students from public high schools (12th graders)*	*Thailand*	*Thai*	*428 (Structural validity, internal consistency, construct validity - convergent & known groups). A sub-sample of 38 (reliability)*	*Random selection of 5 classrooms out of 14*	*99%*
(Runkawatt, 2007)	*FAQ*	*17 to 18 (M=17.46, SD=0.50)*	*67%*	*Students from public high schools*	*Thailand*	*Thai*	*70 (Internal consistency, reliability, construct validity - convergent & known groups)*	*Random selection of 2 classrooms*	*95%*
(Unwin et al., 2008)	FAQ 1	0 to 20 (11% of sample); 20 to 40 (44% of sample); 40 to 60(8% of sample); >60 (11% of sample); other (7%)	33%	First year medical students at the Uniformed Services University of the Health Sciences	Not reported	English?	428 (Construct validity - known groups)	Convenient?	67%
(Wang et al., 2010)	FAQ 1	Group A - (M= 28.63); Group B - (M=18.78)	Not reported	University student nurses including both licensed nurses with work experience and first-year undergraduates with no work experience	China (Taiwan)	Chinese	220, including two sub-groups: Group A - licensed nurses (125) and Group B - undergraduate nursing students (95). (Internal consistency, construct validity - convergent & known groups)	Convenient	84%
(Wang et al., 2010)	FAQ 1	Not reported	Not reported	general geriatricians, a faculty member from the Department of Family Medicine, gerontological experts and a school administrator	China (Taiwan)	Chinese	9 (Content validity)	Convenient	Not reported

Author	Specific questionnaire	Age range (M, SD)	Gender (% female)	Population type	Country	Language	N (measurement property tested)	Sampling method	Response rate
(Nakao et al., 2013)	Knowledge of Aging for Social Workers (version of FAQ)	20 to 70 (M=32, SD=0.5)	90%	Graduate social work students from 35 schools across the country	USA	English?	481(Internal consistency, construct validity - convergent)	Convenient?	Not reported
(Pachana et al., 2013)	FAQ2 -MC	(M=19.9, SD= 5.1)	87%	First year university students	Australia	English?	151 (Cross-cultural validity/measurement invariance)	Convenient	Not reported
(Van der Elst et al., 2014)	Revised FAQ (based on FAQ1 and FAQMH)	Not reported	Not reported	Experts in geriatric and gerontology	Belgium	Dutch	9 (Content validity)	Convenient?	Not reported
(Van der Elst et al., 2014)	Revised FAQ (based on FAQ1 and FAQMH)	18 to 53 (M= 21.1, SD = 4.987)	77%	First and third year nursing students	Belgium	Dutch	1141, including first year students (801) and third year students (340). (Structural validity, internal consistency, construct validity - known groups)	Convenient	72% of the schools and 20% of the students
(Helmes, 2016)	FAQ2 -MC	50 to 94 (M= 70.3, SD=8.88)	69%	Community dwelling older adults	Canada	English?	172 (Cross-cultural validity/Measurement invariance)	Convenient	56%
(Helmes and Pachana, 2016)	FAQ2 -MC	*Canadian sample: 50 to 94 (M=70.3, SD=8.88); Australian sample: 50 to 90 (no details)*	*Canadian sample: 69%; Australian sample: 73.8%*	*Both Canadian and Australian samples: Community dwelling older adults; 50+ for the Australian sample*	*Canada; Australia*	*English?*	*Canadian sample: 172; Australian sample: 195 (Internal consistency, construct validity - convergent)*	*Convenient for both Canadian and Australian samples*	*Canadian sample: 56%; Australian sample: 95%*
(Shiovitz-Ezra et al., 2016)	*FAQ1*	*(M=46, SD=1.6)*	*Approx. 50%*	*Community dwellers, >21, Jewish, and Hebrew speakers*	*Israel*	*Hebrew*	*92 (Structural validity, internal consistency, construct validity - convergent & known groups)*	*Probability sampling*	*52%*
**Fraboni Scale of Ageism (FSA)**									
(Fraboni et al., 1990)	FSA	16 to 65 (M=31.19)	65%	University students, hospital personnel, small businesses, health and education settings	Canada?	English?	231 (Structural validity, internal consistency, construct validity-convergent and known-groups)	Purposive sampling	98% of students; 82% of others
(Fraboni et al., 1990)	FSA	Not reported	50%	High school students	Canada?	English?	120 (Content validity- pilot test)	Convenient?	Not reported
(Fraboni et al., 1990)	FSA	Not reported	Not reported	Psychometrics and clinical psychologists	Canada?	English?	9 (Content validity)	Convenient?	Not reported
(Rupp et al., 2005)	FSA	(M=22.6)	70%	Undergraduates in a public university	USA	English?	353 (Structural validity, internal consistency, construct validity-convergent and known-groups)	Convenient	Not reported
(Rupp et al., 2005)	FSA	(M=22.1)	71%	Undergraduates in a public university	USA	English?	201 (Structural validity)	Convenient	Not reported

**Author**	**Specific questionnaire**	**Age range** (M, SD)	**Gender** (% female)	**Population type**	**Country**	**Language**	**N** (measurement property tested)	**Sampling method**	**Response rate**
(Bodner and Lazar, 2008)	FSA	(M=27.9, SD=7.3)	54%	University students	Israel	Hebrew?	491 (Structural validity, internal consistency, construct validity - known groups)	Convenient	Not reported
(Boudjemad and Gana, 2009)	*FSA*	*Not reported*	*Not reported*	*Bilingual experts knowledgeable of psychology*	*France?*	*French*	*5 (Content validity - adaptation of questionnaire to French)*	*Not reported*	*Not reported*
(Boudjemad and Gana, 2009)	*FSA*	*(M=24.33, SD=8.52)*	*Not reported*	*Not reported*	*France?*	*French*	*30 (Content validity - pilot French translation of original English questionnaire to assess comprehensibility)*	*Convenient?*	*Not reported*
(Boudjemad and Gana, 2009)	*FSA*	*(M=23.17, SD=6.25)*	*Not reported*	*Bilingual students*	*France?*	*French*	*32 (Cross-cultural validity/measurement invariance)*	*Convenient?*	*Not reported*
(Boudjemad and Gana, 2009)	*FSA*	*(M=23.31, SD=7.68)*	*63%*	*University students*	*France?*	*French*	*323 (Structural validity, internal consistency, construct validity - convergent). Sub-sample of 32 (reliability)*	*Convenient*	*Close to 60%*
(Boudjemad and Gana, 2009)	*FSA*	*17 to 74 (M=30.25, SD=13.58)*	*65%*	*University students and older adults*	*France?*	*French*	*284 (Structural validity, construct validity - known groups)*	*Convenient*	*Close to 75%*
(Lin et al., 2010)	FSA	17 to 35 (M= 19.69, SD = 2.99)	74%	First year psychology Anglo-Australian and Asian university students who had been in Australia for less than 5 years	Australia	English	65 (Internal consistency, cross-cultural validity/measurement invariance, construct validity - convergent)	Convenient	Not reported
(Kutlu et al., 2012)	FSA	(M=33.21, SD=13.41)	72%	Students in schools and family members	Turkey	Turkish	231(Structural validity, internal consistency, construct validity - known groups)	Convenient	77%
(Kutlu et al., 2012)	FSA	Not reported	Not reported	Nurse academicians	Turkey	Turkish	12 (Content validity)	Convenient	Not reported
(Kutlu et al., 2012)	FSA	Not reported	Not reported	Not reported	Turkey	Turkish	30 (Content validity - pilot study)	Convenient	Not reported
(Helmes and Pachana, 2016)	*FSA*	*Canadian sample: 50 to 94 (M=70.3, SD=8.88). Australian sample: 50 to 90 (not reported)*	*Canadian sample: 69%. Australian sample: 74%*	*Both Canadian and Australian samples: Community dwelling older adults,*	*Canada & Australia*	*English?*	*Canadian sample: 172 Australian sample: 195 (Internal consistency, construct validity - convergent)*	*Convenient*	*Canadian sample: 56%; Australian sample: 95%*
(Shiovitz-Ezra et al., 2016)	*FSA*	*(M=46, SD=1.6)*	*Approx. 50%*	*Community dwellers, >21, Jewish, and Hebrew speakers*	*Israel*	*Hebrew*	*92 (Structural validity, internal consistency, construct validity - convergent and known groups)*	*Probability sampling*	*52%*

Author	Specific questionnaire	Age range (M, SD)	Gender (% female)	Population type	Country	Language	N (measurement property tested)	Sampling method	Response rate
**Image of Aging Scale (IAS)**									
(Levy et al., 2004)	IAS	Not reported	64%	Community dwellers >70 years of age	USA	English?	717 (Content validity-secondary analysis)	Convenient?	95%
(Levy et al., 2004)	IAS	Not reported	50%	>70 years	USA	English?	10 (Content validity)	Convenient	Not reported
(Levy et al., 2004)	IAS	Not reported	60%	>65 years	USA	English?	10 (Content validity)	Convenient	Not reported
(Levy et al., 2004)	IAS	Not reported	Not reported	Experts in gerontology	USA	English?	5 (Content validity)	Convenient	Not reported
(Levy et al., 2004)	IAS	Not reported	Not reported	Not reported	Not reported	Not reported	Not reported (Content validity -pilot test)	Convenient?	Not reported
(Levy et al., 2004)	IAS	(M=66.4, SD=10.9)	40%	Not reported	USA	English?	20 (Reliability)	Convenient	Not reported
(Levy et al., 2004)	IAS	(M=69.1, SD=12.0)	44%	Not reported	USA	English?	68 (Internal consistency, construct validity - convergent)	Convenient	Not reported
(Levy et al., 2004)	IAS	(M=73.0, SD=8.7)	Not reported	Older adults	USA	English?	76 (Construct validity - known groups)	Convenient	Not reported
(Bai et al., 2012)	Self-Image of Aging Scale	Not reported	Not reported	Older adults (15 from urban and 15 from rural areas)	China	Chinese	30 (Content validity)	Not reported	Not reported
(Bai et al., 2012)	Self-Image of Aging Scale	Not reported	Not reported	Older adults	China	Chinese	20 (Content validity)	Not reported	Not reported
(Bai et al., 2012)	Self-Image of Aging Scale	60 to 69 (60%), 70 to 79 (28.5%), 80 to 89 (11.5%)	50%	Community dwelling older adults	China	Chinese	445 (Structural validity, internal consistency, cross-cultural validity/measurement invariance, reliability, construct validity- convergent)	Multistage sampling with stratification	89%
(Fernández-Ballesteros et al., 2017)	IAS	18+ (M=46.98, SD=18.08)	51% and 54% for sub-sample	Community dwellers	Spain	Spanish	1,105 randomly divided into two groups of 552 and 553 participants. (Structural validity, internal consistency, cross-cultural validity/measurement invariance). And sub-sample aged 50+ (n=448) (construct validity - convergent).	Random	Not reported
(Fernández-Ballesteros et al., 2017)	IAS	(M=43,5, SD=10.18)	49%	Volunteer members of the Spanish Assoc. of Geriatrics and Gerontology	Spain	Spanish	325 (Structural validity, internal consistency, cross-cultural validity/measurement invariance)	Convenient	Not reported
**Kogan's Attitudes towards Old People Scale (KAOP)**									
(Kogan, 1961)	KAOP	Not reported	Not reported	Experts	USA	English?	Not reported (Content validity - Scale development)	Not reported	Not reported

**Author**	**Specific questionnaire**	**Age range** (M, SD)	**Gender** (% female)	**Population type**	**Country**	**Language**	**N** (measurement property tested)	**Sampling method**	**Response rate**
(Kogan, 1961)	KAOP	Not reported	17%	Introductory university class in psychology	USA	English?	482 (Internal consistency, construct validity - convergent & known groups)	Convenient	Not reported
(Hicks et al., 1976)	KAOP	*Not reported*	*Not reported*	*University students, undergraduate*	*USA*	*English?*	*69 (Reliability, construct validity - convergent)*	*Random*	*Not reported*
(Wingard, 1980)	KAOP	*Not reported*	*Not reported*	*University students*	*USA*	*English?*	*69 (Structural validity, reliability, construct validity - convergent)*	*Not reported*	*Not reported*
(Hilt, 1997)	Revised-KAOP	(M=29)	59.30%	Local news producers	USA	English?	87 (Internal consistency, criterion validity)	Probability sampling: market size used in a stratified random sample	41%
(Söderhamn et al., 2000)	KAOP	18 to 24 (32%); 25 to 49 (59%); 50+ (9%)	94%	University students, nurses and other caregivers working in care homes or local hospitals	Sweden	Swedish	319 (Structural validity, internal consistency, construct validity - known groups)	Convenient	100%
(Lambrinou et al., 2005)	KAOP	Not reported	Not reported	Experts in nursing education	Greece	Greek	4 (Content validity - questionnaire adaptation to Greek context)	Convenient	Not reported
(Lambrinou et al., 2005)	KAOP	17 to 48, (M=21)	83%	First and third year nursing students in two universities	Greece	Greek	390 including first year students (121) and final year students (78) (Structural validity, internal consistency, construct validity - known groups)	Convenient?	100%
(Ogiwara et al., 2007)	KAOP	Not reported	56.10%	Physiotherapists working in hospitals, clinics, nursing homes	Japan	Japanese	181 (Structural validity, internal consistency, construct validity - convergent)	Convenient	57%
(Runkawatt, 2007)	KAOP	*Not reported*	*Not reported*	*Experts in geriatrics*	*Thailand*	*Thai*	*3 (Content validity)*	*Convenient*	*Not reported*
(Runkawatt, 2007)	KAOP	*12th grade high school students*	*Not reported*	*Students from public high schools (12th graders)*	*Thailand*	*Thai*	*10 (Content validity - pilot test readability and understandability)*	*Convenient*	*Not reported*
(Runkawatt, 2007)	KAOP	*16 to 19 (M=17.2, SD=0.52)*	*60%*	*Students from public high schools (12th graders)*	*Thailand*	*Thai*	*428 (Structural validity, internal consistency, construct validity - convergent & known groups). Of these 38 (reliability)*	*Random selection of 5 classrooms out of 14*	*99%*
(Runkawatt, 2007)	KAOP	*17 to 18 (M=17.46, SD=0.50)*	*67%*	*Students from public high schools*	*Thailand*	*Thai*	*70 (Internal consistency, reliability, construct validity - convergent & known groups)*	*Random selection of 2 classrooms*	*95%*
(Iwasaki and Jones, 2008)	KAOP	*(M=21.6, SD=3.8)*	*54%*	*Undergraduate college students enrolled in counseling psychology*	*USA*	*English?*	*512 (Internal consistency, construct validity - convergent)*	*Convenient*	*Not reported*

**Author**	**Specific questionnaire**	**Age range** (M, SD)	**Gender** (% female)	**Population type**	**Country**	**Language**	**N** (measurement property tested)	**Sampling method**	**Response rate**
(Helmes and Campbell, 2009)	KAOP	*(M=24.3, SD=8.5)*	*68%*	*Undergraduate university students*	*Australia*	*English?*	*60 (Internal consistency, construct validity - known groups)*	*Convenient*	*Unclear*
(Yen et al., 2009)	KAOP	Not reported	Not reported	Experts, including general geriatricians, a faculty member from the Department of Family Medicine, gerontological experts and a school administrator	China (Taiwan)	Chinese	9 (Content validity)	Not reported	Not reported
(Yen et al., 2009)	KAOP	Not reported	49%	Medical and nursing undergraduates at a central Taiwan Medical University	China (Taiwan)	Chinese	275 (Structural validity, internal consistency, reliability)	Convenient	88.40%
(Erdemir et al., 2011)	KAOP	Not reported	Not reported	Experts including a geriatrician, family physician, sociologist, faculty members from social work, dietetics, sports, health management, physical therapy, psychology, communication, nursing departments and a clinical nurse	Turkey	Turkish	12 (Content validity)	Convenient?	Not reported
(Erdemir et al., 2011)	KAOP	Not reported	Not reported	Students	Turkey	Turkish	30 (Content validity - pilot test)	Convenient?	Not reported
(Erdemir et al., 2011)	KAOP	18 to 27 (M=21.76, SD=1.72)	81%	Undergraduate university students who provide health care to the elderly in the future	Turkey	Turkish	594 (Structural validity, internal consistency). And a subsample of 402 (reliability)	Not reported	71%
(Küçükgüçlü et al., 2011)	KAOP	Not reported	Not reported	Experts including a family physician, a faculty member from the Department of Neurology, clinical nurses, and faculty members from the School of Nursing	Turkey	Turkish	7 (Content validity)	Convenient?	Not reported
(Küçükgüçlü et al., 2011)	KAOP	Not reported	Not reported	Nursing students	Turkey	Turkish	30 (Content validity - pilot test)	Convenient?	Not reported
(Küçükgüçlü et al., 2011)	KAOP	18 to 26 (M=20.08, SD=1.6)	82.30%	University students	Turkey	Turkish	237 (Structural validity, internal consistency, reliability)	Convenient	Not reported
(Kiliç and Adibelli, 2011)	KAOP	Working nurses group (M=27.36, SD=4.54); Nursing	Working nurses group (Not reported); Nursing	Third and fourth year university nursing students and hospital nurses	Turkey	Turkish	263, including university students (145) and working nurses (118). (Structural validity, internal consistency, reliability, construct validity - convergent & known groups).	Convenient	Not reported

Author	Specific questionnaire	Age range (M, SD)	Gender (% female)	Population type	Country	Language	N (measurement property tested)	Sampling method	Response rate
		students group (M=20.17, SD=1.40)	students group (100%)						
(Rejeh et al., 2012)	KAOP	Not reported	Not reported	Experts including a geriatrician, sociologist, faculty members from nursing, health management, psychology and nursing departments and clinical nurses	Iran	Persian	20 (Content validity)	Convenient?	Not reported
(Rejeh et al., 2012)	KAOP	Not reported	Not reported	Nursing students	Iran	Persian	20 (Content validity - pilot test)	Convenient?	Not reported
(Rejeh et al., 2012)	KAOP	(M=31.01, SD=5.96)	71%	Nurses working in five different teaching hospitals	Iran	Persian	350 (Structural validity, internal consistency, construct validity - known groups). A subsample of 70 (reliability)	Non-random	97%
(Matarese et al., 2013)	KAOP	Not reported	Not reported	Experts including geriatric physicians, nurses, and psychologists	Italy	Italian	11 (Content validity)	Not reported	Not reported
(Matarese et al., 2013)	KAOP	18 to 54 (M=25, SD=5.9)	69%	Nursing students attending one of two universities in Rome	Italy	Italian	1637 (Structural validity, internal consistency)	Convenient	73%
(Vitman-Schorr et al., 2014)	KAOP	(M=32.57, SD=10.73)	59%	Community dwellers aged 18 to 64 years, living in Tel Aviv	Israel	Hebrew	300 (Structural validity, internal consistency)	Convenient	Not reported
**Reactions to Aging Questionnaire (RAQ)**									
(Gething, 1994)	RAQ	Not reported	Not reported	Nurses undertaking post basic education at University	Australia	English?	60 (Content validity)	Not reported	Not reported
(Gething, 1994)	RAQ	Not reported	Not reported	300 nurses working at acute care hospital (n =157), community (n = 79) and nursing homes (n = 64); 150 from the general community	Australia	English?	450 (Internal consistency)	Purposive	Not reported
(Gething, 1994)	RAQ	Not reported	Not reported	Registered nurses at a hospital	Australia	English?	100 (Structural validity)	Not reported	Not reported
(Gething, 1994)	RAQ	20 to 69	88%	Health professionals including health promotion workers, nurses, psychologists, social workers, doctors and physiotherapists	Australia	English?	531 (Structural validity, internal consistency, construct validity – convergent & known groups)	Purposive	Not reported

Author	Specific questionnaire	Age range (M, SD)	Gender (% female)	Population type	Country	Language	N (measurement property tested)	Sampling method	Response rate
(Netz et al., 2001)	RAQ	45 to 55	100%	Australian born women aged 45 to 55 participating in the Melbourn Women's Midlife Health Project	Australia	English?	381 (Content validity, structural validity, internal consistency)	Random digit telephone dialing	56% at baseline, of which 86% returned RAQ
(Getting et al., 2002)	RAQ	Reported per age group per country	Australia: 88.4%, UK: 93.1%	People who worked in a nursing profession, particularly gerontology	Australia, UK	English?	Australia: 147, United Kingdom: 218 (Construct validity – convergent & known groups)	A proforma developed to maximize uniformity between the 2 countries	70% across the two countries
(Gething et al., 2004)	RAQ	Reported per age group per country	Australia: 88.4%, United Kingdom: 93.1%, Sweden: 86.3%	People who worked in different nursing specialties including community health, orthopedic, intensive care, emergency, oncology. The majority worked in gerontology.	Australia, Sweden, UK (Australia and UK-same sample as Getting 2002)	English, Swedish	Australia: 147, United Kingdom: 218, Sweden: 102 (Structural validity, internal consistency, cross-cultural validity, construct validity - known groups)	Convenient	Average of 78% across 3 countries
(Helmes and Pachana, 2016)	*RAQ*	*Canadian sample: 50 to 94 years (M=70.3, SD=8.88); Australian sample: 50 to 90 years (Not reported)*	*Canadian sample:69%; Australian sample: 73.8%*	*Both Canadian and Australian samples: Community dwelling older adults, 50+for the Australian sample*	*Canada; Australia*	*English?*	*Canadian sample:172; Australian sample: 195 (Internal consistency, construct validity - convergent)*	*Convenient for both Canadian and Australian samples*	*Canadian sample:56%; Australian sample: 95%*
**Tuckman and Lorge Questionnaire (TLQ)**									
(Tuckman and Lorge, 1954)	*TLQ-% responses*	*21 to 51 (M=32.4, SD=7.3)*	*38.30%*	*Graduate students attending a summer class on the psychology of the adult*	*USA*	*English*	*47 (Construct validity - convergent, responsiveness)*	*Convenient*	*Not reported*
(Tuckman and Lorge, 1954)	TLQ -*Yes/No responses*	*Not reported*	*35.5%*	*Class in the psychology of the adult during the regular academic year*	*USA*	*English*	*124 (Construct validity - convergent, responsiveness)*	*Convenient*	*Not reported*
(Axelrod and Eisdorfer, 1961)	TLQ	*Reported per subgroup*	*38%*	*Students attending an introductory psychology university course*	*USA*	*English?*	*280 (Construct validity - known groups)*	*Not reported*	*Not reported*
(Eisdorfer, 1966)	TLQ -*88 most valid items as found in (Axelrod and Eisdorfer, 1961)*	*16 to 23 (M=18.8)*	*38.5%*	*Introductory psychology course*	*USA*	*English?*	*182 (Construct validity - known groups)*	*Convenient?*	*Not reported*

**Author**	**Specific questionnaire**	**Age range** (M, SD)	**Gender** (% female)	**Population type**	**Country**	**Language**	**N** (measurement property tested)	**Sampling method**	**Response rate**
(Eisdorfer, 1966)	TLQ *-137 original (Tuckman and Lorge, 1953)*	*16 to 23 (M=19.2)*	*44.2%*	*College students*	*USA*	*English?*	*147 (Construct validity - known groups)*	*Convenient?*	*Not reported*
(Hicks et al., 1976)	*Tuckman-lorge Attitude Scale (modified version by (Axelrod and Eisdorfer, 1961))*	*Not reported*	*Not reported*	*university students, undergraduate*	*USA*	*English?*	*69 undergraduate students (35 in introductory developmental psychology and 34 in adult development and aging) (Construct validity - convergent). A subsample of 32 of those not enrolled in the ageing class (Reliability)*	*Random*	*Not reported*
(Wingard, 1980)	*Tuckman-Lorge Attitude Scale(modified version by (Axelrod and Eisdorfer, 1961))*	*Not reported*	*Not reported*	*University students*	*USA*	*English?*	*69 (secondary data analysis) (Structural validity, reliability, construct validity - convergent)*	*Not reported*	*Not reported*
(Helmes and Campbell, 2009)	TLQ *(1952)*	*24.3(8.5)*	*68%*	*University undergraduate*	*Australia*	*English?*	*60 (Internal consistency, construct validity - known groups)*	*Convenient*	*90 volunteered, 30 excluded due to missing data*

Italics are used to signal articles that assessed more than one scale
? indicates that the entry was implicitly reflected in the record. For example, 'English?' indicates that the authors of this article interpreted that this was the language used in the record based on the information provided in the record.

Supplementary Table 2
Characteristics of the different ageism scales

Author	Specific questionnaire	Form of administration (e.g. Self or other administered, online or face-to-face)	Subscale/s # of items	Response options	Range of scores/scoring	Original language	Available translation
**Aging Perceptions Questionnaire (APQ)**							
(Barker et al., 2007)	APQ	Not reported	35 items, including 7 subscales (timeline chronic, timeline cyclical, consequence positive, consequence negative, emotional representations, control positive and control negative). 3 items were removed after validation. Also included an identity sub-scale	5-point Likert scale from 1 (strongly disagree) to 5-(strongly agree) for all subscales except for the identity which had a yes/no response format	0-17 range per subscale except for the identity sub-scale, which had a 0-100 range	English?	Not reported
(Ingrand et al., 2012)	APQ	Self-administered, paper and pencil. In case of difficulty in reading or writing responses, a third party could assist	32 items, including 7 sub-scales, each comprising 3 to 5 items (timeline chronic, timeline cyclical, consequence positive, consequence negative, emotional representations, control positive, and control negative)	5-point Likert scale ranging from 1 (strongly disagree) to 5 (strongly agree). For the negative control dimension only, the response scale is reversed	1 to 5 per dimension	English / Irish?	French
(Sexton et al., 2014)	APQ	Computer assisted home interview	32 items, including 7 sub-scales	5-point Likert scale ranging from 1 (strongly agree) to 5 (strongly disagree)	Not reported	English?	Not reported
(Sexton et al., 2014)	Brief Ageing Perceptions Questionnaire (B-APQ)	Self-administered? paper and pencil?	17 items, including 5 sub-scales	5-point Likert scale ranging from 1 (strongly agree) to 5 (strongly disagree)	Not reported	English?	Not reported
(Slotman et al., 2015)	APQ	Self-administered, paper and pencil	32 items, including 7 sub-scales (timeline chronic, timeline cyclical, consequence positive, consequence negative, emotional representations, control positive and control negative)	5-point Likert scale ranging from 1 (strongly agree) to 5 (strongly disagree). The response scale of the control negative dimension is reversed	Not reported	English	Dutch
(Slotman et al., 2015)	Aging Perceptions Questionnaire -Short (APQ - S)	Self-administered, paper and pencil	21 items from the original 32, including the 7 original sub-scales (timeline chronic, timeline cyclical, consequence positive, consequence negative, emotional representations, control positive and control negative)	5-point Likert scale ranging from 1 (strongly agree) to 5 (strongly disagree). The response scale of the control negative dimension is reversed	Not reported	English	Dutch
(Slotman et al., 2015)	B - APQ	Self-administered, paper and pencil	17 items, including 5 sub-scales (timeline chronic, consequence positive, control positive, emotional representations and the combined consequence and control negative dimension)	Control negative dimensions are not reverse coded	Not reported	English	Dutch
(Chen et al., 2016)	APQ	Self-administered? paper and pencil?	49 items, including 8 sub-scales (timeline acute/chronic and cyclical, consequences positive and negative, control positive and negative, emotional representations, identity).	5-point Likert scale from 1 (completely disagree) to 5 (completely agree) for all sub-scales except identity. Certain items are reverse coded	Not reported	English	Chinese

Author	Specific questionnaire	Form of administration (e.g. Self or other administered, online or face-to-face)	Subscale/s # of items	Response options	Range of scores/scoring	Original language	Available translation
(Moghadam et al., 2016)	B-APQ	Self-administered, paper and pencil. For illiterate participants, the questionnaire was read and their answers recorded by the researcher	17 items including 5 subscales	Nor reported	Not reported	English	Persian
(Slotman et al., 2017)	APQ - S	Self-administered, paper and pencil	2 items, including 7 sub-scales, each comprised of three items (timeline - cyclical and chronic/acute, consequence both positive and negative, control both positive and negative, emotional representations)	5-point Likert scale ranging from 1 (totally disagree) to 5 (totally agree). The scale for control negative items is reversed, and responses were recoded so that higher scores were indicative of greater perceived negative control	Not reported	Dutch	Turkish
**Aging Semantic Differential Test (ASD)**							
(Rosencranz and McNevin, 1969)	ASD	Self-administered paper and pencil	32 items grouped into 3 dimensions (Instrumental-Ineffective, Autonomous-Dependent, Personal Acceptability). 3 target objects in the scale: a male between the ages of 20-30, a male between the ages of 40-55, and a male 70-85 years of age judged on the	7-point Likert scale	Mean scores per dimension and object assessed; in this study 2.12-5.62	English	Not reported
(Underwood et al., 1985)	ASD	Self- administered paper and pencil	32 items and two 2 target objects: old person, young person	7-point Likert scale	Not reported	English?	Not reported
(Gekoski et al., 1991)	ASD	Not reported	32 items 3 target objects: the average man or woman in mid-20s, mid-40s, early 70s	7-point Likert scale	Not reported	English?	Not reported
(O'Hanlon et al., 1993)	ASD	Self-administered? Paper and pencil?	32-bipolar-adjectives, grouped into 3 sub-scales (Instrumental-Ineffective; Autonomous-Dependent; Acceptability-Unacceptability)	7-point Likert scale for each item	Not reported	Not reported	Not reported
(Intrieri et al., 1995)	ASD	Self-administered, paper and pencil	32 pairs of bipolar adjectives, grouped into a 3-factor structure (Instrumental-Ineffective; Autonomous-Dependent; Acceptability-Unacceptability).	7-point Likert scale	32-224 with lower scores suggesting a more positive view of older adults.	English?	
(Villar Posada, 1997)	Spanish version of the ASD (Diferencial Semántico del envejecimiento)	Self-administered, paper and pencil	31 pairs of bipolar adjectives obtained from those used in other scales to assess attitudes towards older adults, as well as those arising in interviews with people of different ages conducted by the authors in previous studies	7-point semantic differential scale ranging from a more negative attitude (1) to a more positive attitude (7) for each pair of bipolar adjectives. A score of 4 represented a neutral score	Not reported	English?	Spanish?
(Polizzi and Millikin, 2002)	ASD	Self-administered, paper and pencil	24 polar adjective pairs and the following target objects: ''old man'' and ''old woman''; ''elderly man''and ''elderly woman ''; ''man 70–85 years of age'' and ''woman 70–85 years of age''	7-point Likert scale	Not reported	24-168	English?

Author	Specific questionnaire	Form of administration (e.g. Self or other administered, online or face-to-face)	Subscale/s # of items	Response options	Range of scores/scoring	Original language	Available translation
(Polizzi, 2003)	The Refined Aging Semantic Differential (R-ASD)	Self-administered, paper and pencil	81 bipolar adjectives assigned to the following target objects: man 70–85 years of age and a woman 70–85 years of age. 30 slightly modified pairs were taken from the original 32 pairs of the Aging Semantic Differential.	7-point Likert scale. A score of 4 represented missing data	Not reported	English?	Not reported
(Polizzi, 2003)	R-ASD	Self-administered, paper and pencil	66 bipolar adjective pairs	7-point Likert scale. A score of 4 represented missing data	Not reported	English?	Not reported
(Stewart et al., 2007)	ASD	Not reported	32 bipolar adjectives, including both a 3 and a 4-factor model	5-point Likert scale	Not reported	English?	Not reported
(Iwasaki and Jones, 2008)	R-ASD	Paper and pencil-study 1, computer administration	24 items consisting of 24 bipolar adjectives covering the following sub-scales: Instrumental-Ineffective (I-I), Autonomous-Dependent (A-D), and Personal Acceptability-Unacceptability (PA-D). Questionnaire target objects: males age 20–30, males age 40–45, and males age 70–85.	7-point Likert scale,	Not reported	English?	Not reported
(Iwasaki and Jones, 2008)	R-ASD	Paper and pencil-study 1, computer administration	24 items consisting of 24 bipolar adjectives covering the following sub-scales: Instrumental-Ineffective (I-I), Autonomous-Dependent (A-D), and Personal Acceptability-Unacceptability (PA-D). Questionnaire target objects: older adults	7-point Likert scale,	Not reported	English?	Not reported
(Boudjemad and Gana, 2009)	ASD	Self-administered	26 bipolar items, including 4 factors (Instrumentality of older adults, Autonomy, Acceptability of older people, and Integrity of older people)	Not reported	Not reported	English	French
(Gluth et al., 2010)	ASD	Not reported	32 pairs of bipolar adjectives, including a 3-factor structure (Instrumental-ineffective; Autonomous-dependent; Personal Acceptability-unacceptability)	7-point Likert scale with lower scores representing a more positive attitude on the respective adjective pair	Not reported	English	German
(Gluth et al., 2010)	ASD	Not reported	Final model 26 items, 4 factors	7-point Likert scale with lower scores representing a more positive attitude on the respective pair	Not reported	English	German
(Gonzales et al., 2010)	R-ASD	Self-administered, Paper and pencil format questionnaire	24 items, including the original 3 factors. The questionnaire target object was modified to refer to individuals 65 and over	7-point Likert scale	24-168	English?	
(Carlson, 2015)	R-ASD	Online	117 descriptors from various questionnaires. The questionnaire was considered to reflect the 3 original sub-scales (Personality, Social/Emotional, Physical), but was after testing found to have an alternative 3 sub-scales (Physical stereotypes, Positive Stereotypes and Negative Stereotypes)	11-point Likert scale ranging from 0 to 100% in 10% intervals	Not reported	Not reported	Not reported
(Carlson, 2015)	R-ASD	Online	117 descriptors from various questionnaires, including 21 from Polizzi, 77 from Goldberg, 29 from Robinson and Andersen, 19 from Almerico and Fillmer, 43 from Schmidt and Bolland, and 3 from Boduroglu et al. The questionnaire was considered to reflect the 3 original	5-point Likert scale ranging from none to all	Not reported	Not reported	Not reported

Author	Specific questionnaire	Form of administration (e.g. Self or other administered, online or face-to-face)	Subscale/s # of items	Response options	Range of scores/scoring	Original language	Available translation
			sub-scales (Personality, Social/Emotional, Physical), but was after testing was found to have an alternative 3 sub-scales (Physical stereotypes, Positive Stereotypes and Negative Stereotypes)				
(Gonzales et al., 2017)	ASD	Self-administered, paper and pencil	32 pairs of bipolar adjectives, including a 3-factor structure (Instrumental-ineffective; Autonomous-dependent; Personal acceptability-unacceptability).3 items were dropped based on testing results, and the 3 original factors were not confirmed. Three different meaningful factors emerged instead (Personality and mental health, Societal participation and Physical)	7-point Likert scale with lower scores representing a more positive attitude on the respective pair	Not reported	English?	Mandarin Chinese
**Anxiety about Aging Scale (AAS)**							
(Lasher, 1987)	AAS	Self-administered, paper and pencil	20 items, including 4 subscales, which resulted from the study of an original scale including 84 items	5-point Likert scale	Not reported	English?	Not reported
(Watkins et al., 1998)	AAS	Self-administered, paper and pencil	20 items, including modifications of the original AAS	Not reported	Not reported	English?	Not reported
(Rivera-Ledesma et al., 2007)	Revised- Anxiety about Ageing Scale (R-AAS)	Not reported	The original Anxiety about ageing scale is used as well as the adapted scale for older Mexican adults. Both included 20 items, and identified 4 sub-scales (Fear of older adults, psychological worries, physical appearance, fear of losses)	4-point Likert scale	Not reported	English?	Spanish
(Gao, 2012)	AAS	Self-administered, paper and pencil, and self-administered online	16 items, including 4 subscales, which resulted from the study testing of the original 20 item scale	5-point Likert scale, from strongly agree (1) to strongly disagree (5)	Not reported	English	Chinese
(Koukouli et al., 2013)	AAS	Self-administered? paper and pencil?	20 items, including 4 subscales of five items each (fear of old people, psychological concerns about ageing, anxiety about physical appearance, fear of losses associated with ageing.	4-point Likert scale, from strongly agree (1) to strongly disagree (4)	Not reported	English	Greek
(Sargent-Cox et al., 2014)	AAS	Self-administered, with 84.3% of sample using a paper and pencil format, and 15.7% an online format	20 items, including 4 subscales (fear of old people, psychological concerns, physical appearance, fear of losses)	5-point Likert scale	Not reported	English?	Not reported
**Attitudes to Ageing Questionnaire (AAQ)**							
(Laidlaw et al., 2007)	AAQ	Self-administered, paper and pencil	24 items, including 3 sub-scales (Psychological growth, Psychosocial loss, and Physical change) was the resulting questionnaire from the study, evolving from a 44-item scale with 5 sub-scales	5-point Likert scale ranging from 1 (strongly disagree) to 5 (strongly agree)	Not reported	English	Not reported

Author	Specific questionnaire	Form of administration (e.g. Self or other administered, online or face-to-face)	Subscale/s # of items	Response options	Range of scores/scoring	Original language	Available translation
(Chachamovich et al., 2008)	AAQ	Not reported	22 items, including 3 subscales was the resulting questionnaire from the study, which evolved from an original 44-item scale	4-point Likert scale which evolved from a 5-point Likert scale	Not reported	English	Portuguese?
(Kalfoss et al., 2010)	AAQ	Self-administered, paper and pencil	24 items, including 3 subscales	5-point Likert scale ranging from 1 (strongly disagree) to 5 (strongly agree)	Not reported	English	Norwegian
(Lucas-Carrasco et al., 2013)	AAQ	Self- administered questionnaire	24 items, including 3 subscales with 8 items each (Psychosocial loss, Physical change, Psychological growth)	5-point Likert scale	8-40	English	Spanish
(Shenkin et al., 2014)	AAQ	Self-administered, paper and pencil	24 items, including 3 subscales	5-point Likert scale ranging from 1 (strongly disagree) to 5 (strongly agree)	Not reported	English	Not reported
(Brown et al., 2015)	AAQ	Self-administered	24 items, including 3 dimensions (physical change, psychological growth, psychosocial loss)	5-point Likert scale ranging from 1 (strongly disagree) to 5 (strongly agree)	Not reported	English?	N/A
(Helmes and Pachana, 2016)	AAQ	Self-administered, paper and pencil	Not reported	Not reported	Not reported	Not reported	Not reported
(Marquet et al., 2016)	AAQ	Both Self-administered paper and pencil, and face to face interview (87% of the sample)	24 items, including 3 subscales (psychosocial loss, physical change and psychological growth)	5-point Likert scale ranging from 1 (strongly disagree) to 5 (strongly agree)	8-40 for each subscale	English	French
(Rejeh et al., 2017)	AAQ	Both Self-administered paper and pencil, and face to face interview for illiterates	24 items, including 3 subscales (psychosocial loss, physical change and psychological growth)	5-point Likert scale ranging from 1 (strongly disagree) to 5 (strongly agree)	8-40 points per each dimension	English	Farsi
**Expectations Regarding Aging (ERA)**							
(Sarkisian et al., 2001)	ERA	Not relevant	26 domains of expectations regarding aging were identified through the focus group discussions	Not relevant	Not relevant	English	Not reported
(Sarkisian et al., 2002)	ERA-38	Self-administered, mailed paper and pencil	38 items, including 10 sub-scales (general health, cognitive function, mental health, functional independence, sexual function, pain, sleep, fatigue, urinary incontinence, and appearance). Half of the items concern expectations regarding one's own aging, and the other half concern general expectations of aging	4-point Likert scale ranging from 1 (definitely true) to 4 (definitely false)	0-100 for ERA-38 as well as for each scale	English	Not reported
(Sarkisian et al., 2005)	ERA-38 & shortened ERA-12 (1999 sample)	self-administered, mailed paper and pencil	38 items, which were subsequently reduced to 12 items, including 3 factors with four items each (Factor 1- expectations regarding physical health, Factor 2- expectations regarding mental health, Factor 3- expectations regarding cognitive functioning)	4-point Likert scale ranging from 1 (definitely true) to 4 (definitely false)	Not reported	English	Not reported

Author	Specific questionnaire	Form of administration (e.g. Self or other administered, online or face-to-face)	Subscale/s # of items	Response options	Range of scores/scoring	Original language	Available translation
(Sarkisian et al., 2005)	ERA-38 & shortened ERA-1 (2001 sample)	only reported for reliability testing where paper and pencil forms were mailed	12 items including e factors as identified in the 1999 sample (Factor 1- expectations regarding physical health, Factor 2- expectations regarding mental health, Factor 3-expectations regarding cognitive functioning)	4-point Likert scale ranging from 1 (definitely true) to 4 (definitely false)	Not reported	English	Spanish
(Joshi et al., 2010)	ERA-12	self-administered, paper and pencil?	12 items, including 3 subscales with 4 items each: expectations regarding physical health, expectations regarding mental health, and expectations regarding cognitive function	4-point Likert scale ranging from 'definitely true' to 'definitely false' response options	0-100, with lower scores indicating expectations of decline	English	Not reported
(Beser et al., 2012)	ERA-12	researchers administered questionnaire, paper and pencil?	12 items, including 3 subscales: physical health, mental health, cognitive functions (4 items per subscale)	4-point Likert scale, ranging from 1 (definitely true) to 4 (definitely false)	0-100 per sub-scale, and 0-100 for entire scale	English	Turkish
(Sparks et al., 2013)	ERA-38	36% self-administered & completed on site using paper and pencil, 13% self-administered & returned by post using paper and pencil, and 51% self-administered using an on-line survey format.	38 items including 2 subscales, one called Aging Self-expectations (ASE) with 18 items, and another one called General Aging Expectations (GAE) with 20 items. Analysis were conducted separately for each sub-scale. For ASE, 8 items failed to load, and 3 factors were identified: Factor 1-self-perceived functional health with aging, Factor 2- self-perceived social health with aging, Factor 3- self-perceived sexual function with aging. For GAE, 8 items failed to load and 3 factors were identified: Factor 1-satisfaction contentment with aging, Factor 2-physical function with aging, Factor 3- cognitive function with aging.	4-point Likert scale ranging from 1 (definitely true) to 4 (definitely false). Higher values on the scale indicate more positive expectations	Not reported	English?	Not reported
**Facts on Aging Quiz (FAQ)**							
(Palmore, 1977)	FAQ	Not reported	25 items	True/False	% of correct	English?	
(Klemmack, 1978)	FAQ	Self-administered, paper and pencil	25 items	True/False	% of correct answers and negative and positive bias item	English?	Not reported
(Holtzman and Beck, 1979)	FAQ	Self-administered, paper and pencil	25 items	True/False	Not reported	English?	Not reported
(Miller and Dodder, 1980)	FAQ	Not reported	25 items	Not reported	Not reported	English?	Not reported
(Miller and Dodder, 1980)	Miller-Dodder Revision (MDR)	Not reported	25 items	Not reported	Not reported	English?	Not reported
(Laner, 1981)	FAQ	Self-administered, paper and pencil	25 items	True/False	Not reported for the scale only for samples	English?	Not reported

Author	Specific questionnaire	Form of administration (e.g. Self or other administered, online or face-to-face)	Subscale/s # of items	Response options	Range of scores/scoring	Original language	Available translation
(Luszcz, 1982)	FAQ	Self-administered, paper and pencil	25 items	True/False	% of errors	English	Not reported
(Romeis and Sussman, 1982)	FAQ	Not reported	25 items	Not reported	Not reported	English?	Japanese?
(Matthews et al., 1984)	FAQ	Not reported	25 items (substituting the term Canada in item 19)	True/False	% of errors	English?	English?
(Miller and Dodder, 1984)	FAQ & MDR	Not reported	25 items	Not reported	% of correct	English?	Not reported
(Courtenay and Weidemann, 1985)	FAQ 1	Self-administered? Paper and pencil?	25 items	True/False	% of incorrect	English?	Not reported
(Courtenay and Weidemann, 1985)	FAQ 1	Self-administered? Paper and pencil?	25 items	True/False/Don't know.	% of incorrect, % of 'don't know'	English?	Not reported
(Courtenay and Weidemann, 1985)	FAQ 2	Self-administered? Paper and pencil?	25 items	True/False	% of incorrect	English?	Not reported
(Courtenay and Weidemann, 1985)	FAQ 2	Self-administered? Paper and pencil?	25 items	True/False/Don't know	% of incorrect, % of 'don't know'	English?	Not reported
(Dail and Johnson, 1983, 1986)	FAQ	Not reported	Not reported	Not reported	Not reported	Not reported	Not reported
(McCutcheon , 1986)	Psychological Facts on Aging Quiz (PFA)	Not reported	22 items (3 new items; #6 FAQ; #10 FAQ2; #3 Miller & Doddler FAQ)	True/False	Not reported	English?	Not reported
(Donnelly et al., 1987)	Revised FAQ & Dye and Saddenrath Aging Test	Not reported	25 items from the revised Facts on Aging Quiz and 40 items from the Dye and Sassenrath test FAQ, tested as a combined and as separate scales	Not reported	Not reported	Not reported	Not reported
(Norris et al., 1987)	FAQ	Not reported	25 items including some modifications (e.g. substituting "United States" in statement 19 with "Canadian)	Not reported	Not reported	English?	Not reported
(Kline et al., 1990)	FAQ & Knowledge of Aging and the Elderly (KAE)	Self-administered, paper and pencil	25 items	True/False	Mean number of correct answers per questionnaire	English?	Not reported

Author	Specific questionnaire	Form of administration (e.g. Self or other administered, online or face-to-face)	Subscale/s # of items	Response options	Range of scores/scoring	Original language	Available translation
(Kline et al., 1990)	FAQ & KAE	Self-administered, paper and pencil	25 items	True/False	Mean number of correct answers per questionnaire	English?	Not reported
(Kline and Kline, 1991a)	FAQ and the Knowledge of Aging and the Elderly (KAE)	Self-administered, paper and pencil?	50 items in total including 25 items from the FAQ and 25 items from the KAE.	True/False?	Not reported	English?	Not reported
(Kline and Kline, 1991b)	FAQ & KAE	Not reported	50 items in total including 25 items from the FAQ and 25 items from the KAE. Minor changes were made to reflect Canadian terminology.	Not reported with the exception that 2 KAE items would be scored differently: correct, incorrect	0-25 per scale	Not reported	Not reported
(O'Hanlon et al., 1993)	FAQ	Self-administered, paper and pencil	25	True/False	Not reported	Not reported	Not reported
(O'Hanlon et al., 1993)	KAE	Self-administered, paper and pencil	25	True/False	Not reported	Not reported	Not reported
(Lusk et al., 1995)	FAQ 1	Not reported	25	True/False	Not reported	English?	Not reported
(Lusk et al., 1995)	FAQ 2	Not reported	25	True/False	Not reported	English?	Not reported
(Harris and Changas, 1994)	FAQ 2- Multiple choice (MC)	Not reported	25 items, the scoring of #16 was changed to True; used the term African American instead of Black	Multiple choice with 4 response options	0-25	English?	Not reported
(Harris and Changas, 1994)	FAQ 2- True/False (T/F)	Not reported	25 items, the scoring of #16 was changed to True	True/False	0-25	English?	Not reported
(Harris et al., 1996)	FAQ 1 T/F	Not reported	Not reported. Some modifications to the wording of the items.	True/False	0-25	English?	Not reported
(Harris et al., 1996)	FAQ 1-MC	Not reported	Not reported	Multiple choice with 4 response options	0-25	English?	Not reported
(Kramer et al., 2001)	FAQ-TF	Not reported	25 items	True/False	% of correct	English?	Not reported
(Kramer et al., 2001)	FAQ-MC	Not reported	25 items	Multiple choice	% of correct	English?	Not reported
(Obiekwe, 2001)	FAQ I- TF	Not reported	25 items	True/False	Not reported	English?	Not reported
(Obiekwe, 2001)	FAQ I- MC	Not reported	25 items	Multiple choice	Not reported	English?	Not reported
(Obiekwe, 2001)	FAQ II- TF	Not reported	25 items	True/False	Not reported	English?	Not reported

Author	Specific questionnaire	Form of administration (e.g. Self or other administered, online or face-to-face)	Subscale/s # of items	Response options	Range of scores/scoring	Original language	Available translation
(Obiekwe, 2001)	FAQ II- MC	Not reported	25 items	Multiple choice	Not reported	English?	Not reported
(Pennington, 2001)	FAQ 1-TF	Not reported	25 items with some revisions to account for the context in New Zealand. 16 items maintained following revision	True/False	Not reported	English?	Not reported
(Pennington, 2001)	FAQ 1 (TF/Don't Know format - DK)	Not reported	25 items with some revisions to account for the context in New Zealand. 16 items maintained following revision	True/False/Don't know	Not reported	English?	Not reported
(Seufert and Carrozza, 2002)	FAQ 1 & 2	Self-administered, paper and pencil	50 items including 25 from FAQ1 and 25 from FAQ 2, and including some wording modifications	True/False/Don't know	% of correct	English?	Not reported
(Cowan et al., 2004)	R-FAQ	Not reported	25 items with some modifications, and including15 negative bias questions, 5 positive bias questions, 5 neutral questions	Not reported	Not reported	Not reported	Not reported
(Runkawatt, 2007)	FAQ	Self-administered?, paper and pencil	25 items with 6 items revised in the final model	True/False	Not reported	English	Thai
(Unwin et al., 2008)	FAQ 1	Not reported	Not reported	Not reported	0-25	Not reported	Not reported
(Wang et al., 2010)	FAQ 1	Self-administered, paper and pencil	25 items with some wording modifications	True/False/Don't know	0-25, % of correct	English	Chinese
(Nakao et al., 2013)	Knowledge of Aging for Social Workers (version of FAQ)	Not reported	25 items, including items from part III Facts of the FAQ on Aging Mental Health	5 multiple-choices for response including 'I don't know'	0-25 (possible), 0-22 (obtained by participants)	Not reported	Not reported
(Pachana et al., 2013)	FAQ2 -MC	Not reported	25 items with some modifications	Multiple choice	% of correct	English?	English?
(Van der Elst et al., 2014)	R-FAQ (based on FAQ1 and FAQMH)	Not reported	36 items (FAQ1 and FAMHQ were used as a basis for developing the revised version). 22 items of the original FAQ1 and 12 items from the FAMHQ were used. 2 new items about life expectancy and risk of physical injury were added	True/False/Don't know	Not reported	English?	Dutch
(Helmes, 2016)	FAQ2 –MC	Not reported	25 items with some modifications to the scoring key for 4 items and other changes to meet the Canadian context	Not reported	0-25	Not reported	Not reported
(Helmes and Pachana, 2016)	FAQ2 –MC	Self-administered, paper and pencil?	Not reported	Not reported	Not reported	Not reported	Not reported
(Shiovitz-Ezra et al., 2016)	FAQ 1	Phone	12 out of the original 25 items were used based on face validity	Not reported	Not reported	English	Hebrew
**Fraboni Scale of Ageism (FSA)**							

Author	Specific questionnaire	Form of administration (e.g. Self or other administered, online or face-to-face)	Subscale/s # of items	Response options	Range of scores/scoring	Original language	Available translation
(Fraboni et al., 1990)	FSA	Self-administered, paper and pencil	44 items (out of the 50 original items), including avoidance (10), discrimination (9) and anti-locution constructs (10)	4-point Likert scale from strongly disagree to strongly agree. Responses were scored from 1-5 for negative items and 5-1 for positive items, with unanswered items scored as 3. A high score is indicative of high ageism.	Not reported	English?	Not reported
(Fraboni et al., 1990)	FSA	Self-administered, paper and pencil	29 items (15 items of the 44 original items were dropped during the study to enhance the psychometric properties of the scale). These items corresponded to three different constructs: Antilocution (10) Discrimination (9) and Avoidance (10)	4-point Likert scale ranging from strongly disagree to strongly agree. Responses were scored from 1-5 for negative items and 5-1 for positive items, with unanswered items scored as 3.	Not reported	English?	Not reported
(Boudjemad and Gana, 2009)	FSA	Not reported	23 items, including 3 dimensions (stereotypes, separation, and affective attitude). 9 items were dropped following testing, which results in a new version of the scale called FSA-14.	5-point Likert scale	Not reported	English	French
(Boudjemad and Gana, 2009)	Fraboni Scale of Ageism 14 (FSA-14)	Not reported	14 items, including 3 dimensions	5-point Likert scale	Not reported	English	French
(Rupp et al., 2005)	FSA	Self-administered, paper and pencil?	Originally 29 items corresponding to a 3-factor model: beliefs about older adults (10 items); separation factor (8); affective (4). 6 items were excluded given findings in confirmatory factor analyses, resulting in a 23-item instrument (stereotype, separation, affective)	4-point Likert scale from 1(strongly disagree)-4(strongly agree)	Not reported	English?	Not reported
(Bodner and Lazar, 2008)	FSA	Self-administered, paper and pencil	22 items (out of the original 29 FSA items, including 3 factors: Avoidance (8), Contribution (5), Stereotypes (6)	Not reported	Not reported	English?	Not reported
(Lin et al., 2010)	FSA	Computer?	29 items	4-point Likert scale	Not reported	Not reported	Not reported
(Kutlu et al., 2012)	FSA	Self-administered, paper and pencil	From the original 29 items, 4 were dropped due to low total correlation of the items. The scale included 3 factors: Stereotypes (11), Avoidance (9), Discrimination (5)	4-point Likert scale from 1(strongly disagree)-4(strongly agree)	29-116	English	Turkish
(Helmes and Pachana, 2016)	FSA	Self-administered, paper and pencil	Not reported	Not reported	Not reported	Not reported	Not reported
(Shiovitz-Ezra et al., 2016)	FSA	Phone	24 items, including 3 factors: Positive perceptions of aging, negative perceptions of aging, and contribution to society	6-point Likert scale from 1 (not agree at all) to 6 (totally agree)	24-144	English	Hebrew

Author	Specific questionnaire	Form of administration (e.g. Self or other administered, online or face-to-face)	Subscale/s # of items	Response options	Range of scores/scoring	Original language	Available translation
**Image of Aging Scale (IAS)**							
(Levy et al., 2004)	IAS	Not reported	18 items, including 9 domains (1 positive, 1 negative item)	0-6 scale	0 – 54	English?	English
(Bai et al., 2012)	Self-Image of Aging Scale	Face to face interview	14 items, including 5 domains	5-point Likert scale	Not reported	English	Chinese
(Fernández-Ballesteros et al., 2017)	IAS	Not reported	18 items, including 9 domains (1 positive, 1 negative item)	7-point Likert scale	Not reported	English?	Spanish
**Kogan's Attitudes toward Old People (KAOP)**							
(Kogan, 1961)	KAOP	Not reported	34 items, including two subscales of 17 items each	7 response options ranging from "strongly disagree" to "strongly agree." A score of 4 was assigned to items left blank	Not reported	English?	Not reported
(Hicks et al., 1976)	KAOP	Self-administered? paper and pencil?	34 items	Not reported	Not reported	Not reported	Not reported
(Wingard, 1980)	KAOP	Self-administered? paper and pencil?	Not reported specifically though mention that minor changes were made to the questionnaire to reflect Canadian culture	Not reported	Not reported	English?	Not reported
(Hilt, 1997)	Revised Attitudes towards Old People Scale (R- KAOP)	Self-administered, paper and pencil?	22 items kept from the original Kogan scale of 34 items	Likert scale ranging from strongly disagree (1) to strongly agree (7). Items left without response (4)	Not reported	English?	Not reported
(Söderhamn et al., 2000)	KAOP	Self-administered questionnaire	34 items, including17 negative items and 17 positive items	Likert scale with 5 response categories	Not reported	English?	Swedish
(Lambrinou et al., 2005)	KAOP	Self-administered, paper and pencil	34 items, including two subscales of 17 items each (one reflecting negative sentiments towards older people and the other one positive)	Likert scale with 6 consecutive alternative response categories	Not reported	English	Greek
(Ogiwara et al., 2007)	Attitudes Towards the Elderly Scale	Self-administered paper and pencil	34 items, including two subscales, though the study identified 3 factors	Likert scale ranging from strongly disagree (1) to strongly agree (7). Items left without response (4)	34-238	English	Japanese
(Runkawatt, 2007)	KAOP	Paper and pencil	34 items, including 17 matched positive/negative pairs of statements about older adults	7-point Likert scale ranging from strongly disagree (1) to strongly agree (7)	Not reported	English	Thai
(Iwasaki and Jones, 2008)	KAOP	Paper and pencil	34 items, including 17 positive and 17 negative items	7-point Likert scale ranging from strongly disagree (1) to strongly agree (7)	Not reported	English?	Not reported
(Iwasaki and Jones, 2008)	KAOP	Paper and pencil	Only 17 positive items from the original 34 items,	7-point Likert scale ranging from strongly disagree (1) to strongly agree (7)	Not reported	English?	Not reported

Author	Specific questionnaire	Form of administration (e.g. Self or other administered, online or face-to-face)	Subscale/s # of items	Response options	Range of scores/scoring	Original language	Available translation
(Helmes and Campbell, 2009)	KAOP	Self-administered, and both paper and pencil and online formats used	34 items, including two subscales of 17 items each	7-point Likert scale	Not reported	English?	Not reported
(Yen et al., 2009)	KAOP	Self-administered, paper and pencil?	34 items, including two subscales of 17 items each	Likert scale ranging from strongly disagree (1) to strongly agree (7). Items left without response (4)	34-238 in this study	English?	Not reported
(Erdemir et al., 2011)	KAOP	Self-administered, paper and pencil	34 items, including two subscales of 17 items each (one expressing negative items and the other expressing positive items)	Likert scale, ranging from strongly disagree (1) to strongly agree (6). Items left without response (4)	34-204.	English	Turkish
(Kiliç and Adibelli, 2011)	KAOP	Self-administered, paper and pencil	34 items, including two subscales of 17 items each. After EFA, only 26 items were retained, including a single factor	6-point Likert scale	34-204	English	Turkish
(Küçükgüçlü et al., 2011)	KAOP	Self-administered, paper and pencil	34 items, including two subscales of 17 items each	Likert scale ranging from strongly disagree (1) to strongly agree (7)	34–238; in this study 74-224	English	Turkish
(Rejeh et al., 2012)	KAOP	Self-administered, paper and pencil	34 items, including two subscales of 17 items each (one expressing negative items and the other expressing positive items)	Likert scale ranging from strongly agree (1) to strongly disagree (7). Items left without response (4)	34-238; in this study 42-188	English	Persian; (article also mentions Norwegian, Greek, Japanese, Chinese, Turkish)
(Matarese et al., 2013)	KAOP	Self-administered paper with supervision	34 items, including 17 items with items worded negatively and 17 items worded positively	Likert scale ranging from strongly agree (1) to strongly disagree (7). Items left without response (4)	34-238	English?	Italian (article also mentions Turkish, Greek, Japanese)
(Vitman-Schorr et al., 2014)	KAOP	Self-administered, paper and pencil	34 items, including two subscales of 17 items each; 5 factors identified in this study	6-point Likert scale ranging from strongly agree to strongly disagree	76-195	English	Hebrew
**Reactions to Ageing Questionnaire (RAQ)**							
(Gething, 1994)	RAQ	Not reported	35 original items, which were reduced to 30 following testing. The scale was found to include 6 distinct factors including anxiety about the future, physical wellbeing, psychological wellbeing, denial of ageing, isolation, activity.	6-point scale ranging from agree to disagree	Not reported	English?	Article mentions translations into Arabic, Italian, Polish, Vietnamese
(Netz et al., 2001)	RAQ	Self-administered, paper and pencil	27 items with 5 factors identified through testing	6-point Likert scale ranging from agree to disagree	Not reported	English?	Not reported
(Getting et al., 2002)	RAQ	Self-administered, paper and pencil	27 items, including three factors (negativity about ageing, anticipated personality attributes of older age, positivity about ageing)	6-point Likert scale on which respondents indicate how much they agree or disagree with each statement	Not reported	English?	Not reported

Author	Specific questionnaire	Form of administration (e.g. Self or other administered, online or face-to-face)	Subscale/s # of items	Response options	Range of scores/scoring	Original language	Available translation
(Gething et al., 2004)	RAQ	Self-administered? Paper and pencil	27 items, with three factors identified through testing: negativity about growing older, perceptions of personality characteristics in older age, and positive attributed perceived to be related to aging	6-point Likert ranging from 1 (agree very much) to 6 (disagree very much)	Possible scores range between 27 and 162 with a higher score indicating more positive attitudes.	English	Swedish
(Helmes and Pachana, 2016)	RAQ	Self-administered, paper and pencil	Not reported	Not reported	Not reported	Not reported	Not reported
**Tuckman-Lorge Questionnaire (TLQ)**							
(Tuckman and Lorge, 1954)	TLQ-% responses	Not reported	A pre-intervention questionnaire including the original 137 statements from the Tuckman & Lorge questionnaire regarding physical changes with age, mental deterioration, activities and interests, family relationships, conservatism, insecurity, etc. A post-intervention, shorter questionnaire including 30 statements from the original questionnaire and 10 questions from another questionnaire about the older worker.	0-100%	Not reported	English	Not reported
(Tuckman and Lorge, 1954)	TLQ-Yes/No responses	Not reported	A pre-intervention questionnaire including the original 137 statements from the Tuckman & Lorge questionnaire. A post-intervention, shorter questionnaire including 30 statements from the original questionnaire and 10 questions from another questionnaire about the older worker.	Yes-No	Not reported	English	Not reported
(Axelrod and Eisdorfer, 1961)	Tuckman and Lorge Attitudes towards Older People	Paper and pencil	137 statements classified into 13 categories: Conservatism, Activities and Interests, Financial, Physical, Family, Personality Traits, Attitude Toward the Future, Best Time of Life, Insecurity, Mental Deterioration, Sex, Interference, and Cleanliness. Subjects were instructed to apply the statements to different target ages: 35-year-olds, 45-year-olds, 55-year-olds, 65- year-olds, or 75-year-olds.	Yes/No	% reporting yes (0-100)	English?	Not reported
(Eisdorfer, 1966)	TLQ-88 most valid items as found in (Axelrod and Eisdorfer, 1961)	Paper and pencil	88 items with subjects instructed to apply the statements to different target age groups, including 35-year-olds, 45-year-olds, 55-year-olds, 65- year-olds, or 75-year-olds.	Yes/No	Mean Yes responses	English?	Not reported
(Eisdorfer, 1966)	TLQ-137 original (Tuckman and Lorge, 1953)	Paper and pencil	137 items with subjects instructed to apply the statements to different target age groups, including 35-year-olds, 45-year-olds, 55-year-olds, 65- year-olds, or 75-year-olds.	Yes/No	Mean Yes responses	English?	Not reported
(Hicks et al., 1976)	Tuckman-lorge Attitude Scale (modified version by (Axelrod and Eisdorfer, 1961))	Not reported	96 items	Not reported	Not reported	Not reported	Not reported

Author	Specific questionnaire	Form of administration (e.g. Self or other administered, online or face-to-face)	Subscale/s # of items	Response options	Range of scores/scoring	Original language	Available translation
(Wingard, 1980)	Tuckman-Lorge Attitude Scale(modified version by (Axelrod and Eisdorfer, 1961))	Not reported	Not reported	Not reported	Not reported	Not reported	Not reported
(Helmes and Campbell, 2009)	TLQ (1952)	Both paper and pencil and web-based self-administration	Not reported	Yes/No	Not reported	English?	Not reported

? indicates that the entry was implicitly reflected in the record. For example, 'self-administered?' indicates that the authors of this article interpreted that this questionnaire was self-administered based on the information provided in the record
